# Transcriptomic analysis of nickel exposure in *Sphingobium* sp. ba1 cells using RNA-seq

**DOI:** 10.1038/s41598-017-08934-7

**Published:** 2017-08-15

**Authors:** M. Volpicella, C. Leoni, C. Manzari, M. Chiara, E. Picardi, E. Piancone, F. Italiano, A. D’Erchia, M. Trotta, D. S. Horner, G. Pesole, L. R. Ceci

**Affiliations:** 1 Department of Biosciences, Biotechnologies and Biopharmaceutics, University of Bari, Bari, Italy; 2 IBIOM-CNR, Institute of Biomembranes, Bioenergetics and Molecular Biotechnologies, Bari, Italy; 3 Department of Biosciences, University of Milan, Milan, Italy; 4 IPCF-CNR, Institute for Chemical-Physical Processes, Bari, Italy

## Abstract

Nickel acts as cofactor for a number of enzymes of many bacteria species. Its homeostasis is ensured by proteins working as ion efflux or accumulation systems. These mechanisms are also generally adopted to counteract life-threatening high extra-cellular Ni^2+^ concentrations. Little is known regarding nickel tolerance in the genus *Sphingobium*. We studied the response of the novel *Sphingobium* sp. ba1 strain, able to adapt to high Ni^2+^ concentrations. Differential gene expression in cells cultured in 10 mM Ni^2+^, investigated by RNA-seq analysis, identified 118 differentially expressed genes. Among the 90 up-regulated genes, a cluster including genes coding for nickel and other metal ion efflux systems (similar to either cnrCBA, nccCBA or cznABC) and for a NreB-like permease was found. Comparative analyses among thirty genomes of *Sphingobium* species show that this cluster is conserved only in two cases, while in the other genomes it is partially present or even absent. The differential expression of genes encoding proteins which could also work as Ni^2+^-accumulators (HupE/UreJ-like protein, NreA and components of TonB-associated transport and copper-homeostasis systems) was also detected. The identification of *Sphingobium* sp. ba1 strain adaptive mechanisms to nickel ions, can foster its possible use for biodegradation of poly-aromatic compounds in metal-rich environments.

## Introduction

The genus *Sphingobium* is notable for the capacity of many of its members to degrade poly-aromatic hydrocarbons^1^, including the insecticide hexachlorocyclohexane^2^. This genus and three closely related genera: *Novosphingobium*, *Sphingomonas* and *Sphingopyxis* were, until a few years ago, classified in the single α-Proteobacteria genus *Sphingomonas*, which included bacteria characterized by the presence of glycosphingolipids in their outer membranes^3^. However, phylogenetic analyses of 16S rRNA gene, together with detailed characterization of fatty acid, glycosphingolipid and polyamine profiles led to their separation into the aforementioned sub-genera^4^. These bacteria are often detected in clinical equipment and specimens, urban water plumbing systems, contaminated soils and waste-water, and in plants^5, 6^. Several lines of preliminary evidence suggest that members of these four genera can grow in the presence of high concentrations of metal ions. *Novosphingobium* sp. PP1Y was reported to grow in the presence of several metal ions (2.5 mM Ni^2+^, 10 mM Pb^2+^, 10 mM Cu^2+^ and 5 mM Zn^2+^)^7^. The strain *Sphingobium* SA2, isolated from mercury contaminated soil, was shown to be capable of reducing mercuric ions, thanks to the expression of a mercuric reductase enzyme^8^. *Sphingobium cupriresistens*, isolated from a copper mine, was reported to tolerate high concentrations of Cu^2+^ ^5^. Members of the genus *Sphingomonas* were detected among bacteria growing in copper polluted environments^9, 10^. A *Sphingopyxis* strain was identified among bacteria living in mining soils, which is able to adapt to high concentrations of Sb^3+^, through oxidation to the less toxic Sb^5+^ ^11^. We recently isolated a novel *Sphingobium* strain (sp. ba1) able to grow in the presence of high concentrations (up to 20 mM) of NiCl_2_. Sequencing of its genome allowed the identification of several genes coding for nickel-dependent enzymes, such as glyoxalase and urease, and proteins potentially involved in efflux-mediated resistance mechanisms^12^.

Nickel is an essential component of several eukaryotic and prokaryotic metallo-enzymes^13, 14^. Due to its employment in many industrial applications, waste-waters from industrial plants often contain millimolar concentrations of Ni^2+^ that are toxic for many organisms^15^. Molecular mechanisms for Ni^2+^ homeostasis, based on uptake, accumulation and efflux systems have been identified in bacteria including *Escherichia coli*, *Cupriavidus metallidurans*, *Helicobacter pylori* and *Achromobacter xylosoxidans* (reviewed in refs 14–17). Here we use the RNA-seq approach^18^ to analyze the response of the *Sphingobium* sp. ba1 strain to high concentrations (10 mM) of nickel ions. Transcriptomic data show the differential expression of about one-hundred and twenty genes, most of which are up-regulated and encode proteins such as membrane proteins and other components of metal efflux systems, enzymes involved in oxidative stress responses (catalases, peroxidases) and signal transduction systems. Interestingly, the up-regulation of several genes annotated as involved in copper ion homeostasis as well as genes encoding proteins belonging to active import systems, might indicate the presence of novel bioaccumulation mechanisms for the homeostasis of nickel ions in *Sphingobium*. A comparative genomic analysis was also carried out to detect whether other *Sphingobium* species can adapt to extracellular high concentrations of nickel ions.

The identification of adaptive mechanisms in the *S. sp* ba1 strain, besides providing insights into the general responses to nickel ions in prokaryotes, may assist in the development of *Sphingobium* bacteria for biodegradation of xenobiotic compounds in metal-rich environments.

## Results

### Cell growth

**Figure 1:**
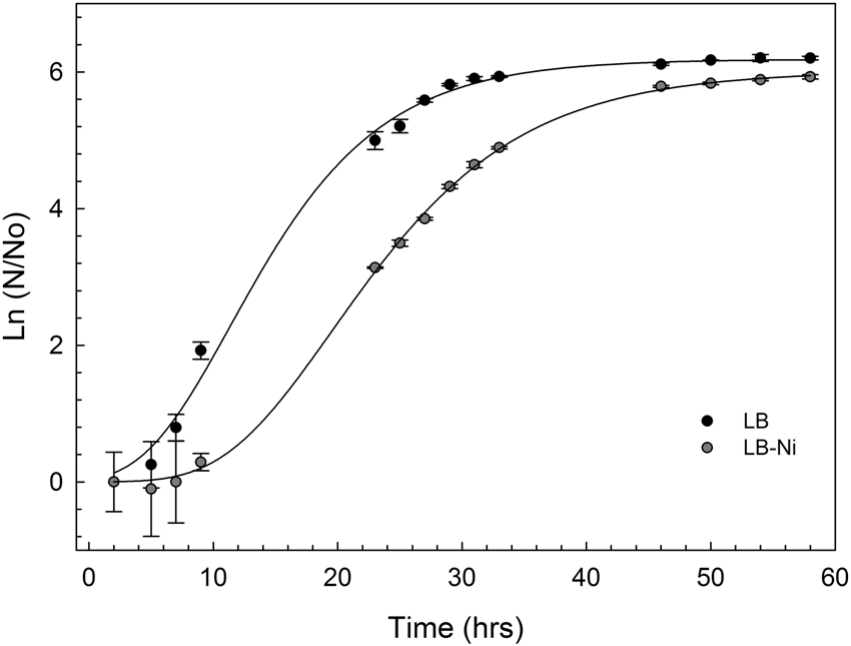
Growth curves of *Sphingobium* sp. ba1 cells. Cell cultures were grown at 30 °C for 60 hours in LB broth and kanamycin, with or without 10 mM NiCl_2_ (both cultures were in triplicate). Optical density of cultures of *Sphingobium*. sp. ba1 in LB and LB-Ni media are shown as a function of growth time.

Single colonies of *S. sp* ba1 cells were inoculated in either LB or LB-Ni media at 30 °C with shaking for 60 hours. Cell growth was evaluated by measuring optical density at 600 nm (OD^600^) (Fig. 1). Growth curves of the two cultures show that cells grown in Ni^2+^ enriched medium have a relatively longer lag-phase, potentially required for the establishment of adaptive mechanisms. The log-phase slope and the stationary state level for the LB-Ni growth curve are only slightly reduced compared to LB-culture, indicating an effective bacteria adaptation to nickel ions.

### RNA sequencing

RNA-seq analysis was performed on three biological replicates of distinct rRNA-depleted RNA preparations, for each growth condition. After quality check and trimming steps, an average of 2621408 and 1641466 reads were obtained for control and LB-Ni cultured cells respectively (Table S1 in Supplementary Materials). RNA-seq data have been deposited in the ArrayExpress database at EMBL-EBI (www.ebi.ac.uk/arrayexpress) under accession number E-MTAB-5828.

Alignment of RNAseq reads against the 87 contigs of the reference genome^12^ was performed using the GSNAP program^19^, and taking into account only uniquely mapped read pairs (in the correct orientation and according to the strand oriented protocol used). This strategy allowed us to map about 90% of fragments on specific contigs (Table S1). Analysis with Cuffdiff^20^, identified 118 significantly differentially expressed genes (p-adjusted < 0.05) (Table S2).

### Transcriptomic analyses

In comparison with controls, Ni^2+^-adapted cells show significant up-regulation of 90 and down-regulation of 28 protein coding genes. While many of these genes (71) are located on the largest contig of the assembly (JPPQ01000069 of 3,212,693 bp), 33 differentially expressed genes are located on contig JPPQ01000083 (600 Kb). A Chi squared test considering the total numbers of genes on these contigs (3489 and 628, respectively) shows a highly significant enrichment of differentially expressed genes on JPPQ01000083 (p-value = 9.436e-06).

Manual refinement of functional annotations of differentially expressed genes was performed using information from the Conserved Domains Database (CDD)^21^ or a combination of protein BLASTP, Pfam and Phyre2 (Table S2). Functional enrichment based on gene ontology, reported in Table 1, shows strong enrichment of terms including transport (GO:0006810), transporter-activity (GO:0005215), membrane (GO:0016020), copper/iron binding (GO:0005507 and GO:0008199), as well as terms related to redox-activity and degradation of oleate (GO:0050151, GO:0006631 and GO:0016491) for the up-regulated genes. Down-regulated genes show strong enrichment of terms related to motility/assembly of the flagellum (GO:0009288 and GO:0001539).

**Table 1:** Functional Enrichment analysis.

GoTerm	Description	Fold-Enrichment	Adj-pvaue (Bonferroni)
**UP-regulated**			
GO:0006810	transport	3.84	7.3747E-07
GO:0005215	transporter-activity	3.92	1.2806E-06
GO:0016020	membrane	2.34	5.7559E-04
GO:0016491	oxidoreductase-activity	1.89	1.4066E-02
GO:0004872	receptor-activity	3.27	3.2456E-04
GO:0005507	copper-ion-binding	12.42	2.7258E-07
GO:0006631	fatty-acid-metabolic-process	10.76	1.2688E-04
GO:0055085	transmembrane-transport	2.3	3.9829E-02
GO:0050151	oleate hydratase activity	35.88	6.1196E-06
GO:0004096	catalase-activity	17.94	1.1758E-04
GO:0020037	heme-binding	3.71	1.5839E-02
GO:0007165	signal-transduction	2.44	4.7196E-02
**DOWN-regulated**			
GO:0016020	membrane	3.26	7.3136E-04
GO:0009288	bacterial-type-flagellum	56.16	2.3805E-12
GO:0004872	receptor-activity	5.06	3.1260E-04
GO:0005215	transporter-activity	3.9	1.2597E-03
GO:0006810	transport	3.56	2.0069E-03
GO:0003735	structural-constituent-of-ribosome	9.56	2.2281E-04
GO:0006412	translation	9.56	2.2281E-04
GO:0009296	flagellum-assembly	>200	0.0000E + 00
GO:0005198	structural-molecule-activity	34.56	7.1217E-05
GO:0001539	ciliary-or-flagellar-motility	9.77	1.3763E-04
GO:0005840	ribosome	3.48	3.2735E-03

GO terms showing significant over-representation in differentially expressed genes sets, with associated counts and adjusted p-values.

### Genes showing differential expression in Ni^2+^ adapted cells

**Figure 2:**
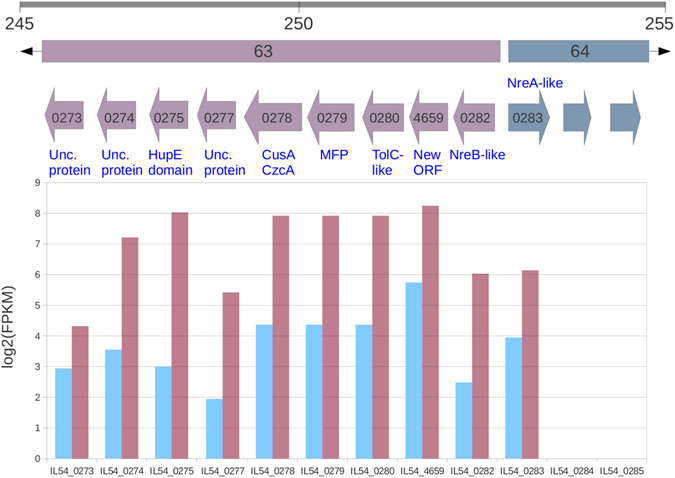
Arrangement and differential expression of genes in the cluster associated with EuGene putative operon n. 63. Gray bar indicates the relative position (in Kb) of the differentially expressed cluster in the JPPQ01000083 contig. Putative operons are indicated by colored bars with black arrow heads. Colored arrows represent genes (identified by the numeric part of their accession number). The name of the encoded protein is given above or under the genes, according to their orientation. The lower panel shows log expression level (FPKM) of genes under control (blue) and LB-Ni (purple) culture conditions.

Several genes likely involved in the export and homeostasis of nickel ions constitute a contiguous cluster (IL54_0273-IL54_0283) in the JPPQ01000083 contig (Fig. 2). IL54_0273-IL54_0282 are annotated on the negative strand, show no expression in LB culture and are highly induced in the presence of nickel. These genes were annotated as a putative operon (number 63) by EuGene. IL54_0283 lies on the positive strand, divergently transcribed with respect to the aforementioned genes, showing some transcription in control conditions and significant induction in nickel-rich media. The encoded polypeptide shows high similarity to NreA-like metal-sensitive transcriptional repressor of the CsoR family^22^. The first gene of the putative operon 63 (IL54_0282) encodes a 418 amino acid polypeptide showing 64% identity with *nreB*, a Major Facilitator Superfamily (MFS) member of *Achromobacter xylosoxidans* ^23^ (accession number AAA72441).

Polypeptides encoded by the central genes of operon 63 (IL54_0278-IL54_0280) are homologous to components of metal ion efflux systems such as CnrCBA (cobalt and nickel resistance) of *Alcaligenes eutrophus* ^24^, NccCBA (nickel-cobalt-cadmium resistance) in *A. xylosoxidans* ^25^ and CznABC (cadmium-zinc-nickel resistance) in *Helicobacter pylori* ^26^ (see Table S2 for further details).

Our original genome annotation^12^ recovered a predicted ORF on the forward strand (IL54_0281) showing convergent transcription towards IL54_0282. However, directional RNA-Seq data showed no evidence of expression of this ORF, but indicated basal expression of the negative strand in the region in control conditions and increased expression in Ni-rich medium. Manual inspection revealed a putative ORF (IL54_4659) of 333 nt, with a GTG initiator, located on the reverse strand, and showing high identity to ORFs present in two *S*. *yanoikuyae* isolates. The encoded polypeptide, like the NreA-like transcriptional repressor encoded in the same cluster (Fig. 2), shows a high percentage of His residues (10%) and shares a similar expression pattern.

5′-RACE analyses identified a single transcription start site for IL54_4659 in both control and LB-Ni cells, located 57 nucleotides upstream of the GTG starting codon, while the same methodology indicated that in LB-Ni cells the IL54_0282 (*nreB*) transcript initiates thirteen nucleotides upstream of the start site predicted by RNA-seq data, indicating that IL54_0282 and IL54_4659 are independently regulated.

RT-PCR experiments showed good agreement with RNA-Seq regarding induction of all of genes in this cluster (Figure S1).

The aforementioned gene cluster is entirely conserved in the genomes of *S. yanoikuyae* SHJ (contig JFFT01000049) and ATCC_51230 (contig AGZU01000009) (Figure S2), while in the other *Sphingobium* species the cluster is absent or incomplete (Table S3). Nucleotide sequence similarities between orthologous genes from this cluster genes in *Sphingobium* sp. ba1, *S. yanoikuyae* SHJ and ATCC_51230 are in the range 89–100% (Table S4).

A second cluster of 6 genes upregulated in Ni-rich medium and representing a probable operon, is also located on contig JPPQ01000083. This cluster consists of ORFs annotated as encoding: a NAD-dependent aldehyde dehydrogenase (NAD-dpn ALDH), a Zn-dependent alcohol dehydrogenase (Zn-dpn AdhP), copper resistance proteins CopA and CopB, a protein of the MFS family and a putative metal-binding protein (IL54_0078, IL54_0079, IL54_0080, IL54_0081, IL54_0082 and IL54_0083 respectively). Additionally, IL54_2275 annotated as coding for CopC, a bacterial protein that binds one copper ion per molecule in the periplasm (Pfam PF04234), is also significantly upregulated in Ni-rich medium.

Interestingly, various putative components of TonB-associated active transport systems were identified among genes differentially expressed in Ni^2+^-rich growth conditions. IL54_0463, the first gene of a predicted operon of three up-regulated genes (Table S2, operon n. 432), encodes a TonB dependent receptor-like protein. The upregulated genes IL54_2990 and IL54_2991, respectively, encode a 205 amino acid polypeptide containing a C-terminal TonB domain, and a 792 amino acid putative iron-uptake protein containing a ligand-gated channel domain (CDD:238657). IL54_3057 is also up-regulated and encodes a Ton-B dependent receptor-like protein of 671 amino acids. Finally, the up-regulated IL54_1841 gene encodes an uncharacterized protein of 700 amino acids, in which a TonB-dependent ligand-gated channel region can be identified.

An interesting two-component signal transduction system, likely involved in the regulation of transcription of ion responsive systems and encoded by a two gene operon including IL54_0770 (a CopS family metal sensor typically associated with heavy metal resistance efflux systems) and IL54_0771 (an OmpR family response regulator^27^) is also up-regulated in high Ni^2+^ conditions.

A putative function of the other differentially expressed genes is reported in the Supplementary Material as “Supplementary description of differentially expressed genes”.

### Comparative genomics of Ni-adaptation among *Sphingobium* species

**Figure 3:**
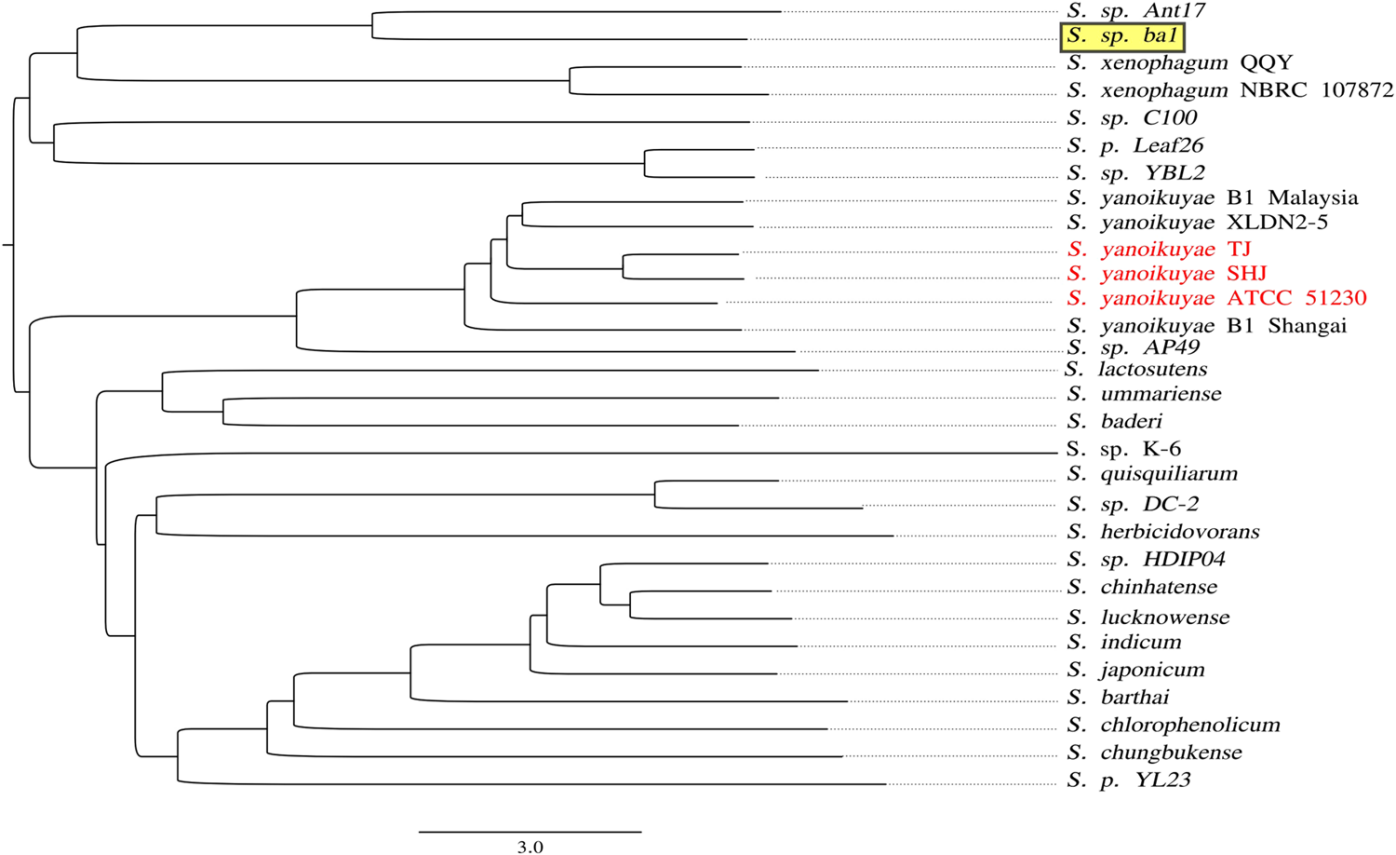
ANIb tree. Phenetic clustering of *Sphingobium* genome sequences based on ANIb (average nucleotide identity based on BLAST analysis). *Sphingobium* sp. ba1 and *Sphingobium* isolates showing a complete and possibly functional Ni^2+^ resistance cluster of genes (see Table S3) have been reported with a yellow background and with red characters, respectively.

Clustering based on ANIb (average nucleotide identity based on BLAST) (Fig. 3) indicates that, of sequenced genomes, *Sphingobium* sp. ba1 genome is most closely related to those of *Sphingobium* sp. strain Ant17^28^ isolate, recovered from Antarctic oil-contaminated soil on the basis of its ability to degrade hydrocarbons, and *S. xenophagum*, another species capable of degrading xenobiotic aromatic compounds^29^. Best reciprocal BLAST matches analyses, recovered 829 putative Clusters of Orthologous Genes represented in all the thirty publicly available *Sphingobium* genomes (Table S5) and constituting the core genome of *Sphingobium*.

The *Sphingobium* sp. ba1 genome contains 184 specific genes (Table S6), not identified in any other available *Sphingobium* genome. Only twenty-two differentially expressed genes (16 up, and 6 downregulated) are part of the *Sphingobium* core genome (Table S2), while 4 of the *Sphingobium* sp. ba1 specific genes are differentially expressed in the course of adaptation to nickel ions (genes IL54_0187, IL54_4442 andIL54_4431 are up-regulated, and IL54_1050 is down-regulated, Table S6), consistent with the hypothesis that adaptive capacity to nickel ions may be restricted to a limited number of isolates.

### Effect of nickel ions on cell morphology

**Figure 4:**
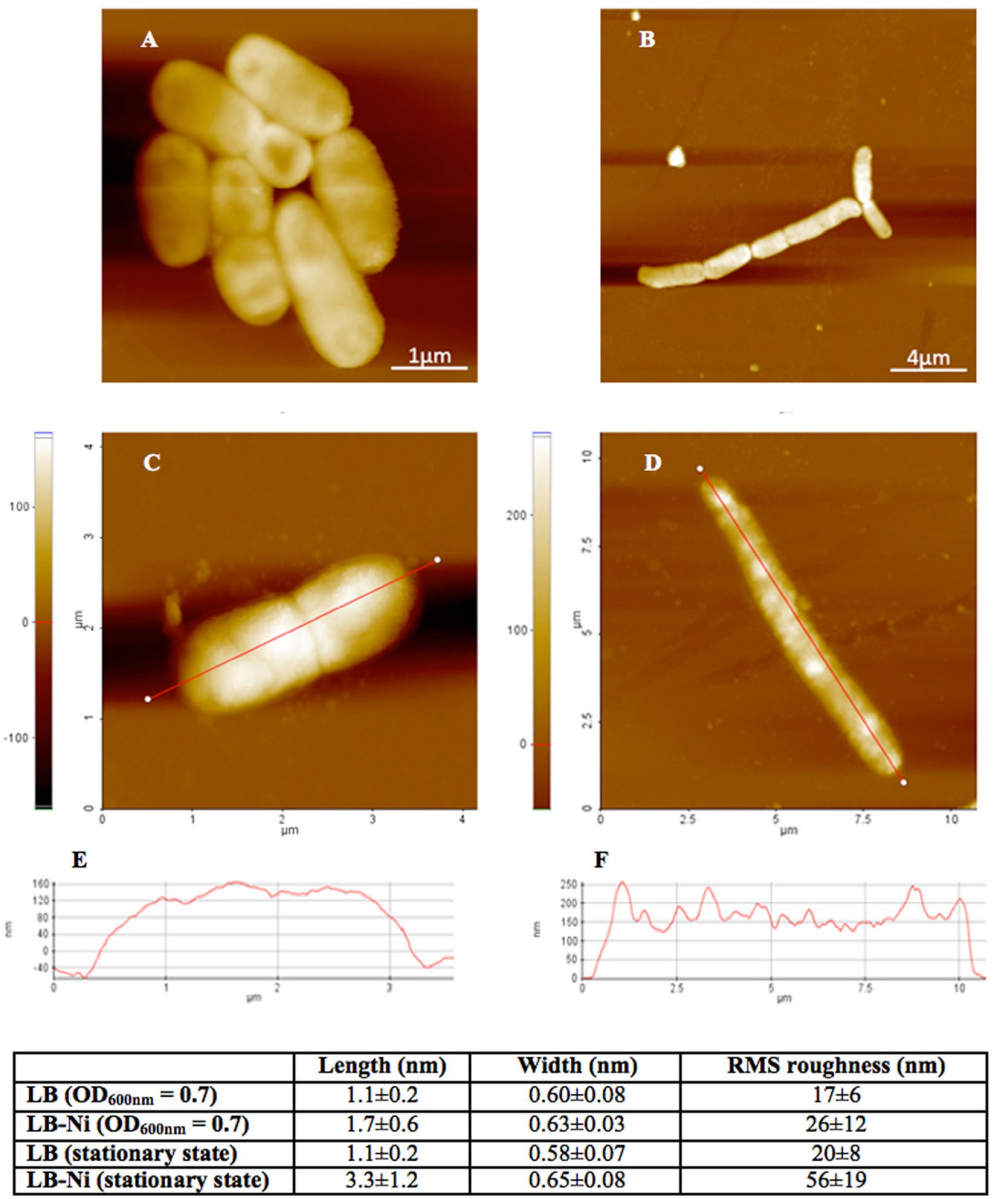
AFM analysis. Comparison of *Sphingobium* sp. ba1 cell morphology cultured in LB (**A**,**B**) and LB-Ni (**D**,**E**) media and harvested at OD_600nm_ = 0.7 (**A**,**D**) and at stationary conditions (**B**,**E**) as obtained by Atomic Force Microscopy (AFM). Height profiles measurements along major cell axes are reported only for cells at stationary state (**C**,**F**). Measurement values are reported in the inset table.

Since adaptation to nickel ions involves the differential expression of numerous membrane associated proteins, cells of *Sphingobium* sp. ba1 were also investigated by AFM to detect possible changes in cell morphology and membrane outward appearance during the adaptive process. Cells were analyzed at mid-log phase and saturation state for both LB and LB-Ni cultures. In both cases, cells appear to have different length and width when exposed to nickel ions, with a larger effect on cell length (Figs 4 and S3). Cell surface also changes in the two growth conditions. Bacterial surface roughness, given as the root mean square RMS value – i.e. the standard deviation of all the height values within the given area - is used as quantitative parameter to evaluate cell surface morphology. The height profiles along major cell axes (given in the table of Fig. 4), show a significant difference in roughness for cells harvested at saturated growth. Cells harvested at half of the exponential phase (OD_600_ = 0.7) show a RMS roughness with a much larger error and a smaller difference.

## Discussion

While numerous genome sequences have been reported for *Sphingobium* species (Table S5), -omics approaches have only recently been applied to the study of their responses to environmental factors^30^. Furthermore, limited attention has been paid to the capacity of members of this genus to adapt to metal ions rich environments. Here we report a RNA-seq based transcriptomic analysis of the adaptive capacity of the *Sphingobium* sp. ba1 strain grown in the presence of 10 mM nickel ions in culture medium. Among the 118 differentially regulated genes, typical bacterial Ni adaptive genes were identified, together with genes considered either to be involved in the homeostasis of other metal ions or in the active transport of nickel ions. The extensive functional analysis, required to verify the possible adaptive mechanisms deduced by the RNA-seq approach, is however out of the scope of this work. Nevertheless, the hypothesized accumulator role for several proteins toward nickel ions is an important subject for further investigation.

Molecular mechanisms for Ni-adaptation in bacteria are mainly based on the efflux of the excess ion. In addition accumulation strategies and reduction to molecular Ni have also been reported^16^. Our data indicate the use of both efflux and accumulation mechanisms in *Sphingobium* sp. ba1 cells, although with some unexpected characteristics.

NreB, a protein of the MFS superfamily, is known to confer resistance to nickel ions in *C. metallidurans* and *A. xylosoxidans* ^23^. In addition, *E. coli* cells transformed with the *A. xylosoxidans nreB* gene showed reduced accumulation of Ni ions, suggesting a role in nickel efflux for NreB. In *Sphingobium* sp. ba1 Ni-exposed cells the *nreB* homolog (IL54_0282) is part of a cluster of up-regulated genes, also containing genes (IL54_0278-IL54_0280) encoding a metal efflux system similar to the CnrCBA, NccCBA and CznABC systems described in other bacteria^24–26^. These efflux systems are made up of an RND (Resistance-Nodulation-cell Division) transporter, a periplasmic MFP (Membrane Fusion protein) and an outer membrane factor (TolC) subunit^15, 16, 31^.

In *Sphingobium* sp. ba1, the action of various Ni-accumulation mechanisms can be hypothesized on the basis of genes observed to be over-expressed in cells grown in Ni-rich medium. For example, the gene showing the highest observed up-regulation (IL54_0275, part of the gene cluster containing the genes for the NreB and RND systems) encodes an uncharacterized protein containing a domain (amino acids 33–185) corresponding to nickel-specific proteins of a secondary metal transporters family (HupE/UreJ)^32^. HupE and UreJ are single component permeases, whose genes are generally part of specific gene clusters for nickel-dependent enzyme systems, [NiFe]-hydrogenases and ureases, respectively^33^. The *Sphingobium* sp. ba1 protein containing the HupE/UreJ domain shows 61% identity with the HupE/UreJ protein identified in *Pseudomonas syringae* (EBI acc. num. S3MDN7). That a nickel permease should be up-regulated in Ni-enriched medium might seem counterintuitive. However, *E. coli* cells expressing either *hupE* or *ureJ* heterologous genes, have been found able to accumulate Ni^2+^ in nickel-rich media^33^. The possibility that some proteins could act as Ni^2+^-sequestration system (either for storage or detoxification) has been proposed for specific proteins, such as SlyD, HspA, Hpn, HypB, UreE^17^, reinforcing the hypothesis of a similar function also for the *S*. sp ba1 HupE/UreJ-like protein.

Genes encoding CopA, CopB and CopC proteins have been found up-regulated in Ni^2+^ -adapted *Sphingobium* sp. ba1 cells. These proteins are known to mediate bacterial resistance to copper^34^ and the *P. syringae* and *Xanthomonas campestris* CopABCD genes, as well as their *E. coli* homologs PcoABCD, have been widely studied and shown to contribute to copper homeostatic mechanisms^35–38^. However, while Ni and Cu bivalent ions are very close in the Irving-Williams series of metal ions for the stability of metal-protein complexes^39^, and have been in some cases found to be interchangeable in metalloproteins^40, 41^, the possibility of the involvement of CopA, CopB and CopC proteins in adaptation to nickel ions has, to our knowledge, not been reported.

The IL54_2797 gene encodes a polypeptide containing a CDD corresponding to a possible CitM H^+^/citrate symporter. In *Bacillus subtilis* CitM can accept the toxic heavy metal ions Zn^2+^, Ni^2+^ and Co^2+^ instead of Mg^2+^ in the metal-citrate complex^42^. Thus, the three polypeptides encoded by the IL54_2795-IL54_2797 operon may constitute a three-component system for the up-take and accumulation of metal-containing anions.

In Gram-negative bacteria TonB-associated transport systems allow the active assimilation of extracellular compounds which are too large to diffuse through porin pores of the outer membrane. TonB-associated active transport systems are composed of outer membrane proteins (TonB-dependent transporters or TBDTs - beta-barrel proteins with high affinity for extracellular metal-chelates) and periplasmic membrane counterparts (TonB, ExbB and ExbD proteins). While outer membrane components (TBDT) are over-expressed in *Sphingobium* sp. ba1 Ni^2+^-adapted cells, inner membrane components (TonB, ExbB and ExbD) do not show expression changes, potentially hinting (once more) at an additional accumulation mechanism. However, the presence of distinct components of TonB dependent transport systems among both up- and down-regulated genes (See Supplementary description of differentially expressed genes) implies that the response of the bacterium to the presence of high concentrations of nickel ions is carried out by a complex coordination of related molecular mechanisms with distinct roles in metal ion homeostasis.

The Ni^2+^-inactivable NreA transcriptional repressor might also act as an accumulator of nickel ions, since its over-expression in the presence of excess Ni^2+^ does not seem to be entirely consistent with its role as a transcriptional repressor that should be effectively inactivated in the presence of excess metal ions. NreA belongs to the CsoR family of sensitive transcriptional repressors (Pfam PF02583), involved in resistance to metal ions. Members of this family bind copper, nickel or cobalt ions via conserved cysteine and histidine residues^43^. In the absence of metal ions, these proteins bind to promoter regions and repress transcription. When bound to metal ions they are unable to bind DNA, leading to transcriptional derepression. The *Legionella pneumophila nreA* gene was demonstrated able to enhance Ni^2+^ resistance in *E. coli* transformed cells^22^.

We note that the genes for two-efflux mechanisms (the RND-efflux system and the NreB protein), together with the permease *hupE*/*ureJ* gene, are tightly clustered in a single genomic region. With the exception of *S. yanoikuyae*, only parts of this cluster can be found in a limited number of *Sphingobium* species (Table S3). The clustering of so many genes for Ni adaptive mechanisms in a such short (about 10 kb) genomic region is quite interesting for functional analysis of this region. Furthermore, the cluster is not present in the genomes of the species most similar to *Sphingobium sp. ba1* in Fig. 3, while in the genomes of most of the *S. yanoikuyae* isolates it is highly conserved, indicating either multiple losses of an ancestral cluster, or horizontal transfer (potentially as part of a mobile element).

The up-regulation of numerous genes coding for proteins of membrane complexes, together with a general cell response to increased osmotic conditions, can also explain the changes in cell morphology occurring at high concentration of nickel ions as observed by AFM. Cells harvested midway through the exponential growth phase (OD_600_ = 0.7) show differences in the RMS roughness and cell length falling within the experimental error. This is probably due to the ongoing cellular division process. Cells harvested under stationary conditions show reduced cellular division and the cell morphology appears more definite, i.e. implying a reduced experimental error. The RMS roughness and cell length of the Ni-exposed cells is more than doubled in comparison to control cells (Fig. 4). The increase in the cell rugosity possibly derives from the presence of many structured spots appearing on the cell surface. Analogous structures observed in other bacteria were attributed to surface proteins and/or lipopolysaccharides (see ref. 44 and references therein). However, exposure to Ni ions also provokes the reduced expression of a number of genes involved in flagellum assembly (Table S2, genes IL54_2820-IL54_2821, IL54_3574 - IL54_3590). Flagella have been described for some *Sphingobium* species^45^ and *Sphingobium sp. ba1* seems likely to possess this structure, as indicated by AFM (Figure S4) and by the presence of a possible complete array of thirty-six flagella-related genes in its genome (data not shown). Nevertheless, the AFM approach failed to prove any direct correlation between reduced expression of genes coding for flagella proteins and alteration of their structure.

In conclusion, the induction of metal ion efflux complexes and the capacity to adsorb nickel ions on membrane associated proteins appear likely to represent the principal mechanisms underlying the adaptation of *Sphingobium* sp. ba1 to high nickel ion concentrations. These mechanisms are likely to be present to varying extents in some other *Sphingobium* species.

*Sphingobium* species have been identified and described essentially for their capacity to degrade polycyclic aromatic hydrocarbons, and therefore they have a remarkable value for bioremediation applications. The additional resistance to metal ions, which has been identified in some species, can extend their application to sites containing different pollutants, allowing easier and more economic remediation processes. The first transcriptomic analysis of a species resistant to high concentrations of a metal ion, allowed the identification of possible general mechanisms adopted by this bacterium to cope with adverse conditions and the description of the distribution of adaptive genes in other sequenced *Sphingobium* genomes. These findings are expected to accelerate the development of bioremediation processes incorporating *Sphingobium* species.

## Materials and Methods

### *Sphingobium* bacterial cultures

*Sphingobium* sp. ba1 has an innate resistance to kanamycin thanks to the presence of a portion of the Tn5 transposon containing the IL54_4593 gene^12^. *Sphingobium* sp. ba1 was grown in either LB broth containing 30 µg/ml kanamycin (LB-kan) or in LB-kan supplemented with 10 mM NiCl_2_ at 30 °C for 60 hours. Both the cultures were grown in three biological replicates.

### RNA preparation

Total RNA was isolated using the hot phenol method. Cells, harvested at mid-log phase, were centrifuged at 6,000 × g for 4 min and the pellet was resuspended in 3 ml of 65 °C pre-warmed lysis solution (0.15 M sucrose, 1% SDS, 10 mM Na-acetate pH 4.7). Thereafter the samples were extracted twice with 3 ml of hot phenol pH 4.3 (65 °C) and once with phenol-chloroform-isoamyl alcohol (125:4:1) pH 4.3. RNA was precipitated with 1/10 volume of 3 M Na Acetate (pH 5.2) and 2.5 volumes of ethanol. After incubation at −80 °C for 15 min RNA was pelleted by centrifugation at 13000 × g for 15 min. Pellets were washed with 1 ml of 70% ethanol, re-centrifuged at 13000 × g for 5 min, washed again with 1 ml of cold absolute ethanol, air-dried and resuspended in 0.1 ml of DEPC-water. Solutions were then treated with RQ1 RNase-Free DNase (Promega) at 37 °C for 1 hour, and RNA precipitated in an equal volume of 4 M LiCl. After incubation at −20 °C for 30 min, RNA was pelleted and washed as described above. The pellet was then dissolved in 0.05 ml of DEPC-water.

RNA quality was checked on a 2100 Bioanalyzer system (Agilent Technologies) using the RNA 6000 Pico assay kit (Agilent Technologies). RIN (RNA Integrity Number) was in the 6–7 range. Absence of genomic DNA contamination was checked by specific amplifications (data not shown).

### RNA sequencing

Directional RNA libraries were prepared using the SureSelect Strand-Specific RNA Library Prep for Illumina Multiplexed Sequencing (Agilent Technologies, version A.3, September 2013) using 4 µg of total RNA after depletion of ribosomal RNA (Ribo-Zero Magnetic kit for Gram negative bacteria (Epicentre)) and purification (RNeasy MinElute Cleanup kit). Libraries were checked with the Agilent DNA 1000 assay 2100 and quantified using the Nano-Drop 3300 Fluorospectrometer (Thermo-Scientific) with the Quant-IT PicoGreen assay kit (Life-Technologies). Equimolar quantities of *Sphingobium* sp. ba1 cDNA libraries were pooled and subjected to 2 × 120 bp paired-end sequencing on the Illumina MiSeq platform.

### Bioinformatics analyses

Read qualities were evaluated using FASTQC (http://www.bioinformatics.babraham.ac.uk/projects/fastqc/) and quality trimming performed using “Trim Galore” (http://www.bioinformatics.babraham.ac.uk/projects/trim_galore) with a quality score threshold of 25. Reads were aligned to the *Sphingobium* sp. ba1 genome using GSNAP^19^ and differential expression analyses performed using CuffDiff^20^. Gene annotations were revised to incorporate expression data using EuGene^46^, protein structure predictions were performed with the Phyre2 suite^47^ and genome alignments were generated using the “progressive alignment” option available in the MAUVE program^48^, with default scoring and parameters (*Sphingobium* genomes analysed with the MAUVE program are reported in Table S5). PfamScan^49^, using both the Pfam-A and Pfam-B domain models were used to assign functional domains to predicted proteins. GO terms were mapped to protein coding genes using a custom script and the Pfam2go file available on the GO consortium website (http://ftp.cbi.pku.edu.cn/pub/database/GO/goa/external2go/pfam2go). Functional enrichment analyses were performed using a custom script, implementing a hypergeometric test with a Bonferroni correction. All against all BLASTP^50^ searches were performed using the BLOSUM80 matrix accepting only best reciprocal hits with e-value < = 1e-5 and where “second-best” hits from the same genome produce bit scores <90% of that associated with the best match. Putative Clusters of Orthologous Genes (COGs) were established as groups of best reciprocal BLAST hits. Core genes were defined as COGs containing single representatives from all genomes considered, and accessory genes as COGs with incomplete representation. ANIb (average nucleotide identity based on BLAST) was calculated with a custom script implementing the algorithm described in Goris *et al*.^51^. The phylogenetic tree was generated using the NJ algorithm implemented in the Neighbor program from the PHYLIP package^52^.

### Real-Time PCR (RT-PCR) analysis

ss-cDNA was prepared from 500 ng of RNA (purified from both LB and LB-Ni cultured cells) using the SuperScript First-Strand Synthesis System (Life Technologies). RT-PCR assays were performed as previously reported^53^ using the pairs of primers indicated in Table S7, with 16 S ribosomal RNA as reference gene on a Quant Studio 6 Flex Real-Time PCR System (Applied Biosystems, Life Technologies), using SYBR Green PCR Master Mix (Life Technologies). Amplification parameters were as follow: hot start at 95 °C for 15 min; 40 amplification cycles (94 °C for 15 sec, 60 °C for 30 sec); dissociation curve step (95 °C for 15 sec, 60 °C for 15 sec, 95 °C for 15 sec). Fluorescence raw data were exported by the Flex Real Time PCR System Software (Applied Biosystems, Life Technologies) and analyzed using the DART-PCR Excel workbook^54^. Actual amplification efficiency values (E) for each amplicon were used to correct Cq values before analyzing these data by the ΔCq method to compare relative expression results. Expression levels were calculated as previously described^53^, using LB-growth as internal calibrator.

### 5′-RACE analysis

For 5′-RACE, single strand cDNA (ss-cDNA) was synthesized starting from 1 μg of total RNA and 1 μL of 2 μM gene specific reverse primers (Sphingo_IL54_4569-R and 5_Sphingo_ IL54_0282-Rr, Table S7), using the SuperScript III (LifeTechnologies) at 70 °C for 15 min. 3′-tailing reaction on the obtained single-strand cDNA was then carried out using terminal transferase (LifeTechnologies) and dCTP according to the supplier instructions. Following, tailed ss-cDNA was amplified with the same gene specific reverse primers used in the ss-cDNA synthesis paired with polyG as forward primer, according to the following amplification program: 35 cycles for 1 min at 94 °C, 1 min at 62 °C (66 °C for IL54_0282 transcript) and 1 min at 72 °C. A final hemi-nested PCR amplification was carried out different gene specific reverse primers (5_Sphingo_914-RA and 5_Sphingo_915-RA, Table S7) paired with polyG as forward primer, according to the following amplification program: 35 cycles for 1 min at 94 °C, 1 min at 62 °C, (65 °C for IL54_0282 transcript) and 1 min at 72 °C. Final amplification products were cloned and sequenced according to standard procedures.

### Atomic Force Microscopy (AFM) analysis of bacterial cells

Bacteria were adhered to a poly-L-lysine covered mica slide as previously reported^55^ by room temperature drying of 15 μL of bacterial suspension in milliQ water (10^7^ CFU/mL). Slides were washed briefly with distilled water, dried, and mounted. All experiments were performed in tapping mode using a PSIA XE-100 atomic force microscope (Park System, Suwon - Korea) equipped with a standard 125 µm long microlever with a force constant of 42 N/m and a resonant frequency of 320 kHz. Height and amplitude images for cells grown in control condition and in presence of 10 mM Ni^2+^ were recorded simultaneously. Measurements were started by scanning a random area of 40 × 40 μm which may contain several bacterial cells. These images were used to evaluate the morphology of bacterial cells and aggregates. The scan size was decreased gradually until aggregates and single cells could be observed clearly. Height images revealed the sample topography and were applied to quantify the morphology of bacterial cells. Randomly selected cells (50 per treatment) were measured and analyzed. Amplitude images were captured to analyze surface features since they have higher sensitivity than height images^56^. Bacterial surface roughness, in terms of the root mean square RMS value (the standard deviation of all the height values within the given area), was another quantitative index used to evaluate the cell surface morphology. Height images were used to calculate the roughness of bacterial surface based on RMS values^57^. The measurements were conducted over two different areas (0.5 × 0.5 μm^2^) on the surface of each cell (20 cells per treatment).

## Electronic supplementary material

Supplementary Material

## References

[1] Pinyakong O, Habe H, Omori T (2003). The unique aromatic catabolic genes in sphingomonads degrading polycyclic aromatic hydrocarbons (PAHs). J Gen Appl Microbiol.

[2] Sangwan N (2013). Reconstructing an ancestral genotype of two hexachlorocyclohexane-degrading Sphingobium species using metagenomic sequence data. Isme J.

[3] Yabuuchi E (1990). Proposals of Sphingomonas paucimobilis gen. nov. and comb. nov., Sphingomonas parapaucimobilis sp. nov., Sphingomonas yanoikuyae sp. nov., Sphingomonas adhaesiva sp. nov., Sphingomonas capsulata comb. nov., and two genospecies of the genus Sphingomonas. Microbiol Immunol.

[4] Takeuchi M, Hamana K, Hiraishi A (2001). Proposal of the genus Sphingomonas sensu stricto and three new genera, Sphingobium, Novosphingobium and Sphingopyxis, on the basis of phylogenetic and chemotaxonomic analyses. Int J Syst Evol Microbiol.

[5] Li L, Liu H, Shi Z, Wang G (2013). Sphingobium cupriresistens sp. nov., a copper-resistant bacterium isolated from copper mine soil, and emended description of the genus Sphingobium. Int J Syst Evol Microbiol.

[6] White DC, Sutton SD, Ringelberg DB (1996). The genus Sphingomonas: physiology and ecology. Curr Opin Biotechnol.

[7] D’Argenio V (2014). Complete sequencing of Novosphingobium sp. PP1Y reveals a biotechnologically meaningful metabolic pattern. BMC Genomics.

[8] Mahbub KR, Krishnan K, Megharaj M, Naidu R (2016). Bioremediation potential of a highly mercury resistant bacterial strain Sphingobium SA2 isolated from contaminated soil. Chemosphere.

[9] Altimira F (2012). Characterization of copper-resistant bacteria and bacterial communities from copper-polluted agricultural soils of central Chile. BMC Microbiol.

[10] Besaury L (2013). Culture-dependent and independent studies of microbial diversity in highly copper-contaminated Chilean marine sediments. Microb Ecol.

[11] Shi Z (2013). Correlation models between environmental factors and bacterial resistance to antimony and copper. PLoS One.

[12] Manzari, C. *et al*. Draft genome sequence of Sphingobium sp. strain ba1, resistant to kanamycin and nickel ions. *FEMS Microbiol Lett* (2014). 10.1111/1574-6968.12618 25288103

[13] Boer JL, Mulrooney SB, Hausinger RP (2014). Nickel-dependent metalloenzymes. Arch Biochem Biophys.

[14] Zhang Y, Rodionov DA, Gelfand MS, Gladyshev VN (2009). Comparative genomic analyses of nickel, cobalt and vitamin B12 utilization. BMC Genomics.

[15] Li Y, Zamble DB (2009). Nickel homeostasis and nickel regulation: an overview. Chem Rev.

[16] Macomber L, Hausinger RP (2011). Mechanisms of nickel toxicity in microorganisms. Metallomics.

[17] Kaluarachchi H, Chan Chung KC, Zamble DB (2010). Microbial nickel proteins. Nat Prod Rep.

[18] Wang SW (2009). orf4 of the Bacillus cereus sigB gene cluster encodes a general stress-inducible Dps-like bacterioferritin. J Bacteriol.

[19] Wu TD, Nacu S (2010). Fast and SNP-tolerant detection of complex variants and splicing in short reads. Bioinformatics.

[20] Trapnell C (2012). Differential analysis of gene regulation at transcript resolution with RNA-seq. Nat Biotechnol.

[21] Marchler-Bauer A (2014). CDD: NCBI’s conserved domain database. Nucleic Acids Res.

[22] Kim HY (2002). Characterization of the Nickel Resistance Gene from Legionella pneumophila: Attenuation of Nickel Resistance by ppk (polyphosphate kinase) Disruption in Escherichia colis. J Microbiol Biotechnol.

[23] Grass G (2001). NreB from Achromobacter xylosoxidans 31A Is a nickel-induced transporter conferring nickel resistance. J Bacteriol.

[24] Liesegang H, Lemke K, Siddiqui RA, Schlegel HG (1993). Characterization of the inducible nickel and cobalt resistance determinant cnr from pMOL28 of Alcaligenes eutrophus CH34. J Bacteriol.

[25] Schmidt T, Schlegel HG (1994). Combined nickel-cobalt-cadmium resistance encoded by the ncc locus of Alcaligenes xylosoxidans 31A. J Bacteriol.

[26] Stahler FN (2006). The novel Helicobacter pylori CznABC metal efflux pump is required for cadmium, zinc, and nickel resistance, urease modulation, and gastric colonization. Infect Immun.

[27] Stock AM, Robinson VL, Goudreau PN (2000). Two-component signal transduction. Annu Rev Biochem.

[28] Adriaenssens, E. M., Guerrero, L. D., Makhalanyane, T. P., Aislabie, J. M. & Cowan, D. A. Draft Genome Sequence of the Aromatic Hydrocarbon-Degrading Bacterium Sphingobium sp. Strain Ant17, Isolated from Antarctic Soil. *Genome Announc* **2** (2014). 10.1128/genomeA.00212-14 PMC3983292 24723703

[29] Stolz A (2000). Description of Sphingomonas xenophaga sp. nov. for strains BN6T and N,N which degrade xenobiotic aromatic compounds. Int J Syst Evol Microbiol.

[30] Zhou, N. A., Kjeldal, H., Gough, H. L. & Nielsen, J. L. Identification of Putative Genes Involved in Bisphenol A Degradation Using Differential Protein Abundance Analysis of Sphingobium sp. BiD32. *Environ Sci Technol* (2015). 10.1021/acs.est.5b02987 26390302

[31] Nies DH (2003). Efflux-mediated heavy metal resistance in prokaryotes. FEMS Microbiol Rev.

[32] Rodionov DA, Hebbeln P, Gelfand MS, Eitinger T (2006). Comparative and functional genomic analysis of prokaryotic nickel and cobalt uptake transporters: evidence for a novel group of ATP-binding cassette transporters. J Bacteriol.

[33] Eitinger T, Suhr J, Moore L, Smith JA (2005). Secondary transporters for nickel and cobalt ions: theme and variations. Biometals.

[34] Cooksey DA (1994). Molecular mechanisms of copper resistance and accumulation in bacteria. FEMS Microbiol Rev.

[35] Huffman DL (2002). Spectroscopy of Cu(II)-PcoC and the multicopper oxidase function of PcoA, two essential components of Escherichia coli pco copper resistance operon. Biochemistry.

[36] Bender CL, Cooksey DA (1987). Molecular cloning of copper resistance genes from Pseudomonas syringae pv. tomato. J Bacteriol.

[37] Rouch D, Camakaris J, Lee BT, Luke RK (1985). Inducible plasmid-mediated copper resistance in Escherichia coli. J Gen Microbiol.

[38] Lee YA, Hendson M, Panopoulos NJ, Schroth MN (1994). Molecular cloning, chromosomal mapping, and sequence analysis of copper resistance genes from Xanthomonas campestris pv. juglandis: homology with small blue copper proteins and multicopper oxidase. J Bacteriol.

[39] Irving H, Williams RJP (1948). Order of stability of metal complexes. Nature.

[40] Foster AW, Osman D, Robinson NJ (2014). Metal preferences and metallation. J Biol Chem.

[41] Foster AW, Pernil R, Patterson CJ, Robinson NJ (2014). Metal specificity of cyanobacterial nickel-responsive repressor InrS: cells maintain zinc and copper below the detection threshold for InrS. Mol Microbiol.

[42] Krom BP, Huttinga H, Warner JB, Lolkema JS (2002). Impact of the Mg(2+)-citrate transporter CitM on heavy metal toxicity in Bacillus subtilis. Arch Microbiol.

[43] Sakamoto K, Agari Y, Agari K, Kuramitsu S, Shinkai A (2010). Structural and functional characterization of the transcriptional repressor CsoR from Thermus thermophilus HB8. Microbiology.

[44] Chao Y, Zhang T (2011). Optimization of fixation methods for observation of bacterial cell morphology and surface ultrastructures by atomic force microscopy. Appl Microbiol Biotechnol.

[45] Garrity, G. S. *et al*. *Bergey’s Manual of Systematic Bacteriology: Volume Two: The Proteobacteria*. Second Edition ed., Springer Science & Business Media: (2006).

[46] Sallet E, Gouzy J, Schiex T (2014). EuGene-PP: a next-generation automated annotation pipeline for prokaryotic genomes. Bioinformatics.

[47] Kelley LA, Mezulis S, Yates CM, Wass MN, Sternberg MJ (2015). The Phyre2 web portal for protein modeling, prediction and analysis. Nat Protoc.

[48] Darling AE, Mau B, Perna NT (2010). progressiveMauve: multiple genome alignment with gene gain, loss and rearrangement. PLoS One.

[49] Mistry J, Finn R (2007). Pfam: a domain-centric method for analyzing proteins and proteomes. Methods Mol Biol.

[50] Altschul SF, Gish W, Miller W, Myers EW, Lipman DJ (1990). Basic local alignment search tool. J Mol Biol.

[51] Kretzschmar U, Schobert M, Gorisch H (2001). The Pseudomonas aeruginosa acsA gene, encoding an acetyl-CoA synthetase, is essential for growth on ethanol. Microbiology.

[52] Felsenstein J (2005). Using the quantitative genetic threshold model for inferences between and within species. Philos Trans R Soc Lond B Biol Sci.

[53] Volpicella M (2014). Rhodobacter sphaeroides adaptation to high concentrations of cobalt ions requires energetic metabolism changes. FEMS Microbiol Ecol.

[54] Peirson SN, Butler JN, Foster RG (2003). Experimental validation of novel and conventional approaches to quantitative real-time PCR data analysis. Nucleic Acids Res.

[55] Italiano F (2012). Changes in morphology, cell wall composition and soluble proteome in Rhodobacter sphaeroides cells exposed to chromate. Biometals.

[56] Pelling AE, Li Y, Shi W, Gimzewski JK (2005). Nanoscale visualization and characterization of Myxococcus xanthus cells with atomic force microscopy. Proc Natl Acad Sci USA.

[57] Camesano TA, Natan MJ, Logan BE (2000). Observation of Changes in Bacterial Cell Morphology Using Tapping Mode Atomic Force Microscopy. Langmuir.

