# Supplementary material for: Transcriptomic analysis of nickel exposure in Sphingobium sp. ba1 cells using RNA-seq
Source: Sci Rep. 2017 Aug 15;7:8262. doi: 10.1038/s41598-017-08934-7 (PMC5557971; doi:10.1038/s41598-017-08934-7)
Supplement: Supplementary file 1 — Supplementary Material [file 41598_2017_8934_MOESM1_ESM.doc]

SUPPLEMENTARY MATERIALS

Transcriptomic analysis of nickel exposure in *Sphingobium* sp. ba1 cells using RNA-seq

M. Volpicella^1,a^, C. Leoni^1,a^, C. Manzari^2^, M. Chiara^3^, E. Picardi^1,2^, E. Piancone^1^, F. Italiano^4^, A. D'Erchia^1,2^, M. Trotta^4^, D.S. Horner^2,3^, G. Pesole^1,2^, L.R. Ceci^2,*^

1) Department of Biosciences Biotechnologies and Biopharmaceutics, University of Bari, Bari, Italy. 2) IBBE-CNR, Institute of Biomembranes, Bioenergetics and Molecular Biotechnologies, Bari, Italy. 3) Department of Biosciences, of Milan, Milan, Italy. 4) IPCF-CNR, Institute for Chemical-Physical Processes, Bari, Italy.

1. These Authors have contributed equally to this work.

*) Corresponding Author:

Institute of Biomembranes, Bioenergetics and Molecular Biotechnologies

National Research Council (CNR)

Via Amendola 165/A

70126 Bari, Italy

Phone: +39 080 5443311; Fax: +30 080 5443317

Email: [**l.ceci@ibiom.cnr.it**](mailto:l.ceci@ibbe.cnr.it)

List of Supplementary Material

Text

Supplementary description of differentially expressed genes

Tables

**Table S1. Sequenced cDNA fragments and their mapping.** The number of sequenced and quality-checked cDNA fragments for each RNA preparation is reported. The number of fragments mapped on the *Shingobium* sp. ba1 genome is also given.

**Table S2. Differentially expressed genes in Ni exposed *Sphingobium* sp. ba1 cells.**

**Table S3. Comparative Genomics of *Sphingobium* sp. ba1 genes IL54_0273- IL54_0283 in complete genomes of *Sphingobium* species.**

**Table S4.** Similarity values between proteins encoded by genes IL54_0273-IL54_0283 (including IL54_4659) in *Shingobium* sp. ba1 contig JPPQ01000083.1 and homologous counterparts in the *S. yanoikuyae* strains ATCC_51230 (contig AGZU01000009) and SHJ (contig JFFT01000049).

**Table S5. Available *Sphingobium* ssp. complete genome sequences.**

**Table S6. *Sphingobium* sp. ba1 specific genes.**

Figures

**Figure S1. RT-PCR analysis of the JPPQ01000083-63 operon genes.**

**Figure S2. MAUVE comparison of IL54_0274-IL54_0283 cluster with a selection of *S. yanoikuyae* genomic regions*.***

**Figure S3. AFM images of a *Sphingobium* sp*.* ba1 cells cultured in LB (first two lines) and LB-Ni media (third and fourth lines).**

**Figure S4. AFM images of a *Sphingobium* sp. ba1 cell cultured in LB medium.**

Supplementary description of differentially expressed genes

*I.* Transport and homeostasis

- 1. *Genes coding for Ni^2+^ export systems and proteins involved in Ni^2+^ homeostasis.*

The last three genes of the putative operon 63 (IL54_0277, IL54_0274 and IL54_0273) (Table S2) encode an uncharacterized protein of 140 amino acids (lacking any possible trans-membrane domain), an uncharacterized protein with two possible trans-membrane helices, and another uncharacterized protein devoid of possible trans-membrane domains, respectively.

Other two up-regulated genes which may encode proteins possibly involved in Ni2+ homeostasis are IL54_1860 and IL54_1862. They are annotated as coding for two putative membrane proteins, which seem to share some common characteristics. Pfam search with both the polypeptides, indeed, matched with the RcnB family (Pfam database accession number: PF11776), a periplasmic protein required for the homeostasis of nickel and cobalt ions, in correspondence of amino acids 99-149 (for IL54_1860) and 179-221 (for IL54_1862). In addition, Phyre2 analysis indicates that the C-termini of both proteins, **for about seventy amino acids,** show structural analogies with the C-terminal domain, of the effector protein CigR from *Salmonella enterica* (PDB 4EW5).

- 1. *Genes coding for proteins involved in the homeostasis of copper ions.*

Also the IL54_2270 and IL54_2271 genes code for other CopB and CopA proteins, respectively. These two genes are predicted to form a two-gene operon. It must be underlined, however, that the proposed CopB protein encoded by IL54_2270 has a similarity level of only 25% with the *Pseudomonas* counterpart, and Pfam search failed in matching the protein with any protein family.

Transcription analysis also allowed the identification in the JPPQ01000011 contig of 1716 bp of a coding region for part of a CopA multicopper oxidase protein.

The protein IL54_0192 is annotated as Cation transport ATPase. According to the CDD dabase, it fits with the ATPase-IB1 family for copper and silver translocation (CDD:273664).

- 1. *Genes coding for proteins involved in the homeostasis of other metal ions.*

The up-regulated genes IL54_2795, IL54_2796 and IL54_2797, organized in a putative operon, are annotated as coding for hypothetical proteins although CDD analysis suggest that IL54_2795 encodes the periplasmic component of an ABC-type Fe3+ transport system, while IL54_2796 likely encodes an outer membrane channel and IL54_2796 a CitM H+/citrate symporter. CitM mediated transport is an active process sustained by protons or sodium ion electrochemical gradients. The IL54_2797 gene encodes a polypeptide containing a CDD corresponding to a possible CitM H+/citrate symporter. In *Bacillus subtilis* CitM can accept the toxic heavy metal ions Zn^2+^, Ni^2+^ and Co^2+^ instead of Mg^2+^ in the metal-citrate complex [1]. Thus, the three polypeptides encoded by the IL54_2795-IL54_2797 operon may constitute a three-component system for the up-take and accumulation of metal-containing anions.

The up-regulated IL54_3183 gene encodes a hypothetical protein of 115 amino acids containing a region corresponding to a UrcA protein. In *Caulobacter crescentus*, expression of this gene is highly induced in response to uranium, but not to other heavy metals [2]. BLAST analysis showed that similar proteins are also encoded among *Sphingobium*, *Sphingomonas* and *Novosphingobium* genera.

IL54_3276 encodes a 159 amino acid long bacterioferritin. This kind of protein, beside to be involved in the homeostasis of iron ions, has also been identified as a stress related protein [3] and it is also able to interact with different metal ions as Co^2+^ and Zn^2+^ [4-6].

A gene coding for an uncharacterized protein in which a conserved domain corresponding to an outer membrane receptor, possibly involved in iron transport, can also be detected among down-regulated genes (IL54_1050).

- 1. *Genes coding for subunits of the TonB-associated transport system.*

The other genes of the operon n 463 (Table S2) containing the gene IL54_0463 coding for a TonB- dependent receptor-like protein, encode respectively a hypothetical protein of 188 amino acids, in which 5 trans-membrane helices can be predicted according to the TMpred tool (IL54_0464), and a putative iron uptake protein (PiuB) (IL54_0465).

Another over-expressed protein possibly associated to TonB is IL54_4442. It is a 98 amino acid protein, highly homologous to the C-termini of typical outer membrane channel proteins associated with TonB.

The contig JPPQ01000059 of about 1.2 Kb contains an upregulated sequence corresponding to the first 250 amino acids of a TonB-depedent siderophore receptor of about 715 amino acids (currently, there is no annotation for this sequence in the NCBI database).

Other putative components of TonB-dependent transport systems, including the gene IL54_2290, a TonB-dependent receptor-like protein of 676 amino acids, as well as IL54_2004 encoding an 847 amino acid TonB-depedent receptor-like protein, are down-regulated under excess Ni2+ growth conditions (Table S2).

- 1. *Genes for the HAE RND system, for the efflux of hydrophobic and amphiphilic compounds.*

Another over-expressed protein containing a TolC region is encoded by the IL54_0187 gene. It belongs to an operon of four genes. The first two genes (IL54_0184 and IL54_0183), which are not significantly differentially expressed, code for an outer membrane protein and a large conserved bacterial protein of 1396 amino acids, respectively. The third gene (IL54_0185) codes for an over-expressed L-Ser hydratase, an enzyme classified in the gluconeogenesis cellular process.

Significant up-regulation of a probable operon (IL54_0580-IL54_0582) encoding components of a typical HAE-RND efflux system for hydrophobic and amphiphilic compounds[7,8] is also observed. The role of this efflux system in *S*. sp. ba1 in the presence of high concentrations of Ni ions, could be related to the general stress condition of the bacterium and the requirement of efflux systems specific for toxic compounds.

*II.* Oxidative-Reductive processes

Oxido-reductive enzymes are over-expressed in response to high concentrations of Ni ions. **The JPPQ01000087 contig contains the partial sequences of two genes forming a single operon, according to EuGene analysis, with a potential role in the oxidation of fatty acids. The first gene (IL54_0657) is located in the 5’-side of the contig. It shows high similarity with the C-terminal part of genes coding for 3-hydroxyacyl-CoA dehydrogenase (3HDCH). The second gene, which is not annotated in NCBI but whose presence is confirmed by transcriptional data, codes for a polypeptide longer than 450 amino acids, whose stop codon is outside the contig. Pfam search allows to classify the polypeptide within the MCRA (Myosin-cross-reactive antigen) family (Pfam PF06100). This protein in *Bifidobacterium breve* was shown to be a FAD-dependent fatty acid hydratase, over-expressed in response to heat and solvent stresses [9]. The two genes are present in other *Sphingobium*, *Novosphingobium* and *Sphingomonas* species.**

**IL54_4431 encodes a DyP-type (dye decolorizing peroxidase) family domain 31 containing protein and is the third gene of a predicted three gene operon up-regulated under excess Ni^2+^ conditions. The second gene of the operon (IL54_4430) encodes an uncharacterized protein of 205 amino acids. While Pfam searches did not recover significant matches, Phyre 2 highlighted a strong structural homology (99.2% confidence) with a 33 kD catalase-related protein from *Mycobacterium avium* (PDB 3E4W).**

Other over-expressed catalases are encoded by the genes IL54_1589 (annotated as a putative transposon, but containing a region corresponding to the conserved domain CDD:163708 of Catalase-like heme binding proteins) and IL54_1653 (which contains the same conserved domain). A possible catalase (IL54_0881) can also be identified among genes down-regulated in high Ni^2+^ growth conditions.

IL54_2303 codes for a 109 amino acid long hydroxylase belonging to the 2OG-Fe [10] oxygenase family, also found for the polypeptide encoded by IL54_1747 (see *Regulation of gene expression*). These enzymes typically catalyse the oxidation of organic substrates [11].

IL54_3810 codes for a 499 amino acid long methylmalonate-semialdehyde dehydrogenase. This enzyme, active in the degradation of branched chain amino acids, can be involved in either intermediary metabolism and detoxification [12].

*III.* Regulation of gene expression

The IL54_1746 gene codes for a transcription factor of the GntR family. In mycobacteria a copper responsive GntR protein able to act as global repressor of about three hundreds proteins, among which 37 membrane-associated transport proteins, has been identified [13]. Upstream of the IL54_1746 gene, the gene for a hypothetical protein of 309 amino acids and containing a region corresponding to a member of the 2OG-Fe [10] oxygenase superfamily is present (IL54_1747). The two genes are overlapped for four nucleotides and constitute a single operon located on the minus strand.

The IL54_0798 gene codes for ArsR, a repressor of the *ars* operon, containing genes involved in the detoxification of bacterial cells from arseniate, arsenite and antimonite [14]. Oxyanions like arsenite and antimonite interact with ArsR and induce its release from the promoter of the *ars* operon [15,16]. The gene is the first of an up-regulated three gene operon. The IL54_0797 gene encodes for a hypothetical protein of 115 amino acids with no conserved domains. The third gene of the operon (IL54_0796) encodes a 457 amino acid long protein containing a DyP-type peroxidase region. In *Mycobacterium tubercolsis* two members of the ArsR-SmtB family of metal-sensing transcriptional repressor were also found sensitive to Ni ions [17].

*IV.* Other processes

1. IL54_0272 codes for an uncharacterized protein of 523 amino acids in which two distinct domains can be recognized. The 50-257 region of the protein corresponds to a reverse transcriptase, while the region encompassing amino acids 365-490 corresponds to a trypsin domain. Blast search highlighted the presence of an identical polypeptide in *Sphingobium yanoikuyae* ATCC51230 (ac. number K9CQF6). Homologous sequences among other *Sphingobium* species have not be identified. In the Uniprot (http://www.uniprot.org) database the protein is annotated as involved in the “RNA-dependent DNA replication” Biological process.
2. IL54_0539 encodes a possible acetyl-CoA synthetase of 647 amino acids (GO Biological process: metabolic process), containing nickel ions as cofactor [18]. It may represent a possible cytoplasmic storage tool of excess nickel ions [19]. Up-regulation of this gene has been already reported in *Pseudomonas aureoginosa* grown on media rich in ethanol and acetate [20].
3. A possible transport protein, component of an ABC-type transport system is encoded by the IL54_0678 gene. It is a polypeptide of 401 amino acids containing four trans-membrane domains (according to TMpred). Pfam search shows that a first domain of about 240 amino acids can be classified as a MacB-like periplasmic core domain (Pfam PF12704) sharing structural similarities with the periplasmic domain of the AcrB multidrug efflux transporter.
4. IL54_0951 codes for a protein of 255 amino acids containing an OmpA-like domain, a **peptidoglycan binding domain similar to the C-terminal domain of outer-membrane protein OmpA, found in** several types of bacterial membrane [21].
5. The up-regulated gene IL54_1032 codes for an uncharacterized protein of 184 amino acids containing the domain CDD 273591 for an intracellular protease (PfpI family). In the description of the conserved domain it is reported that in *Bacillus subtilis* a member of this family is expressed in response to stress events. (GO Biological process: metabolic process).
6. The IL54_1658 gene codes for an uncharacterized protein in which part of a conserved domain corresponding to the NADB Rossmann domain for dinucleotide binding [22] is present. (GO Biological process: fatty acid metabolic process).
7. IL54_2731 codes for a protein of 146 amino acids, annotated as ferredoxin. It also contains a region corresponding to proteins of the functionally uncharacterized DUF326 family, characterized by a cysteine-rich four helical bundle.
8. The IL54_2853 gene codes for a 220 amino acid long outer membrane protein W, OmpW (Pfam PF03922).
9. The IL54_3467 gene encodes for a protein of the ApbE family, involved in thiamine biosynthesis (GO Biological process: protein flavinylation). In *Salmonella enterica* it has been shown that its deficiency has negative effects on Fe-S cluster metabolism [23].
10. The IL54_4207 genes is annotated as coding for a putative beta-lactamase of the class A.
11. Uncharacterized proteins
12. IL54_0179 gene encodes for a 122 amino acid protein containing a PilZ domain (amino acids 32-100). This domain binds to cyclic diguanosine monophosphate (C-di-GMP), a typical bacterial second messenger, which at high concentrations reduces cell motility, favouring the establishment of the multicellular state of biofilms [24,25]. In *Borrelia burgdorferi* it has been shown that PlzA (a protein containing the PilZ domain) is involved in maintaining cell motility and virulence [25].
13. The IL54_0733 gene encodes for a 73 amino acid long polypeptide. The sequence is at the beginning of the contig on the minus strand and the encoded polypeptide seems as the N-terminal region of a longer polypeptide. Nevertheless the sequenced region contains a sequence corresponding to the conserved NADB Rossmann domain for dinucleotide binding [22] (see also the IL54_1658 gene in the “Other processes” section).
14. The gene IL54_0911 codes for an uncharacterized protein of 243 amino acids belonging to the DUF541 family. This family in bacteria has an unknown function, and its proteins are thought to be located in the periplasm of outer membrane.
15. IL54_0989 codes for protein classifiable within the uncharacterized bacterial protein family DUF892. The protein also contains a dinuclear metal binding motif.
16. The IL54_1269 gene codes for an uncharacterized protein of 130 amino acids, particularly rich in proline (20%) and alanine (15.4%) residues. Pfam search does not identify any possible family corresponding to this protein. Blast search, however, identified some polypeptides in *Sphingobium* species, annotated as Fe-S oxidoreductases (UniProt ac. number N1MGF5 and A0A086P9P6) with similarity levels around 60%.
17. IL54_1413 codes for a protein of 148 amino acids containing an EF-hand calcium binding motif. This domain pair is composed of two helices connected by a twelve-residue calcium-binding loop. It is the most common calcium-binding motif in proteins [26]. Interestingly an homologous protein in *Schistosoma mansoni* has been shown able to interact with nickel ions [27].
18. IL54_1842 codes for a 263 amino acid protein containing a conserved domain of unknown function (DUF4198) of 221 amino acids. The IL54_1842 protein has a trans-membrane region (6-30). In addition, Phyre2 structural analysis identifies for the IL54_1842 protein a highly structured region of about 200 amino acids (41-254) corresponding to two IgG-like domains of filamin-a. The protein encoded by the IL54_1843 gene shows a possible transmembrane domain (3-21) and the presence of a EF-hand domain pair, as predicted by Phyre2 search. The gene is part of an operon also containing the IL54_1843 gene coding for an uncharacterized protein of 165 amino acids.
19. The IL54_2025 gene encodes for a 63 amino acid long protein of unknown function. Pfam, Blast and Phyre2 searches did not retrieve any hit, even if Blast analysis showed that the protein is highly conserved among *Sphingobium* species.
20. IL54_2636 codes for a protein of 87 amino acids, containing a region corresponding to proteins of the functionally uncharacterized DUF326 family, characterized by a cysteine-rich four helical bundle. A similar region has also been identified in the IL54_2731 polypeptide, annotated as ferredoxin (see Section “Other processes”).
21. IL54_2850 codes for a 120 amino acid long protein. This protein, highly conserved among *Sphingobium* species, shows a high-confidence structure similarity with the *Serratia marcescens* Resistance-Associated Protein RAP1a (PDB3ZF1) as indicated by Phyre2 analysis. RAP proteins in Gram negative bacteria are used for the neutralization of toxic effector proteins produced as growth inhibitors of bacterial competitors [28].
22. IL54_2900 codes for a 400 amino acid long protein. Blast search showed that the protein is homologous to some *Sphingobium* outer membrane proteins of about 600 amino acids. TMPred tool predict four transmembrane domains in the polypeptide sequence.
23. IL54_3074codes for a 191 amino acid long protein belonging to the functionally uncharacterized bacterial protein family DUF924.
24. An up-regulated operon of genes coding for uncharacterized proteins is that constituted by the three genes IL54_3464-IL54_3466. IL54_3464 codes for a protein of 214 amino acids hosting a possible PepSY-associated trans-membrane helix. PepSY indicates the propeptide serving as inhibitor of many proteases, usually bound to the enzyme until it is secreted and can be activated [29]. PepSY-associated trans-membrane helix are a conserved TM helix found in bacteria and archea. IL54_3465 codes for a polypeptide of 177 amino acids in which the FlgD Ig-like domain can be identified. This domains has an immunoglobulin like beta sandwich fold and it is found in the secreted FlgD protein required for the flagellar hook assembling [10]. Also a trans-membrane region (amino acids 7-27) is supposed to be present in this polypeptide.IL54_3466codes for a 302 amino acid protein containing a conserved domain of unknown function (DUF4198) of 226 amino acids. Amino acids 3-23 are predicted to form a trans-membrane region. Structural analysis of the IL54_3466 protein by Phyre2 shows the presence of a highly structured region of about 200 amino acids (63-247) corresponding to two IgG-like domains of filamin-a. Even if filamin is an actin cross-linking protein of about 2600 amino acids, filamin-like regions of reduced length can be found in bacteria (e.g. Blastopirellula marina, NCBI ac. n. WP_002655062.1). The operon organization found for the IL54_3464-IL54_3466 genes is highly conserved among bacteria (not shown).
25. IL54_3615 codes for an uncharacterized protein of 93 amino acids. Pfam, Blast and Phyre2 search did not indicate any possible classification of this protein within already known protein family.
26. IL54_4408 codes for a short over-expressed protein of 38 amino acids, one-third of which are Proline residues. Asp and Glu residues are both over 10% of relative abundance. From Blast analysis it results that the polypeptide can be the C-terminal region of a longer polypeptide of about 60-65 amino acids of unknown function, common to many *Sphingobium* species.
27. The over-expressed IL54_4421 protein is a 126 amino acid long protein, rich in Arg, Ala and Asp residues. No indications from Pfam, Blast and Phyre2 search.
28. IL54_4274codes for an uncharacterized protein of 283 amino acids in which a metal-dependent hydrolase domain can be predicted. It is the part of a two-gene operon, the second member of which is an up-regulated gene (even if below the threshold of the genes reported in Table 3) containing the coding region for an acetyl-transferase.
29. The JPPQ01000083 contig contains two EuGene predicted up-regulated genes which were not present in our original annotation IL54_4663 and IL54_4664 The first gene with a coding region of 291 nucleotides (coordinates: 55138-55428) shows an unusual TTG starting codon, and codes for an uncharacterized protein common to other *Sphingobium* species. The second gene of 402 nucleotides (coordinates: 55752-56153) also codes for an uncharacterized protein highly conserved among *Sphingomonas*, *Novosphingobium* and *Sphingobium* species. It overlaps the NCBI coding sequence IL54_0076 (coordinates: 56073-56153) coding for an hypothetical protein.

REFERENCES

[1] B.P. Krom, H. Huttinga, J.B. Warner, J.S. Lolkema, Impact of the Mg(2+)-citrate transporter CitM on heavy metal toxicity in Bacillus subtilis, Arch Microbiol, 178 (2002) 370-5.

[2] N.J. Hillson, P. Hu, G.L. Andersen, L. Shapiro, Caulobacter crescentus as a whole-cell uranium biosensor, Appl Environ Microbiol, 73 (2007) 7615-21.

[3] S.W. Wang, C.Y. Chen, J.T. Tseng, S.H. Liang, S.C. Chen, C. Hsieh, Y.H. Chen, C.C. Chen, orf4 of the Bacillus cereus sigB gene cluster encodes a general stress-inducible Dps-like bacterioferritin, J Bacteriol, 191 (2009) 4522-33.

[4] R. Janowski, T. Auerbach-Nevo, M.S. Weiss, Bacterioferritin from Mycobacterium smegmatis contains zinc in its di-nuclear site, Protein Sci, 17 (2008) 1138-50.

[5] A.M. Keech, N.E. Le Brun, M.T. Wilson, S.C. Andrews, G.R. Moore, A.J. Thomson, Spectroscopic studies of cobalt(II) binding to Escherichia coli bacterioferritin, J Biol Chem, 272 (1997) 422-9.

[6] N.E. Le Brun, A.M. Keech, M.R. Mauk, A.G. Mauk, S.C. Andrews, A.J. Thomson, G.R. Moore, Charge compensated binding of divalent metals to bacterioferritin: H+ release associated with cobalt(II) and zinc(II) binding at dinuclear metal sites, FEBS Lett, 397 (1996) 159-63.

[7] C. Alvarez-Ortega, J. Olivares, J.L. Martinez, RND multidrug efflux pumps: what are they good for?, Front Microbiol, 4 (2013) 7.

[8] D. Du, Z. Wang, N.R. James, J.E. Voss, E. Klimont, T. Ohene-Agyei, H. Venter, W. Chiu, B.F. Luisi, Structure of the AcrAB-TolC multidrug efflux pump, Nature, 509 (2014) 512-5.

[9] E. Rosberg-Cody, A. Liavonchanka, C. Gobel, R.P. Ross, O. O'Sullivan, G.F. Fitzgerald, I. Feussner, C. Stanton, Myosin-cross-reactive antigen (MCRA) protein from Bifidobacterium breve is a FAD-dependent fatty acid hydratase which has a function in stress protection, BMC Biochem, 12 (2011) 9.

[10] K. Ohnishi, Y. Ohto, S. Aizawa, R.M. Macnab, T. Iino, FlgD is a scaffolding protein needed for flagellar hook assembly in Salmonella typhimurium, J Bacteriol, 176 (1994) 2272-81.

[11] A.J. Prescott, A Dilemma of Dioxygenases (or Where Biochemistry and Molecular Biology Fail to Meet), J. Exp. Bot., 44 (1993) 849-61.

[12] C. Stines-Chaumeil, F. Talfournier, G. Branlant, Mechanistic characterization of the MSDH (methylmalonate semialdehyde dehydrogenase) from Bacillus subtilis, Biochem J, 395 (2006) 107-15.

[13] M. Rao, H. Liu, M. Yang, C. Zhao, Z.G. He, A copper-responsive global repressor regulates expression of diverse membrane-associated transporters and bacterial drug resistance in mycobacteria, J Biol Chem, 287 (2012) 39721-31.

[14] D. Paez-Espino, J. Tamames, V. de Lorenzo, D. Canovas, Microbial responses to environmental arsenic, Biometals, 22 (2009) 117-30.

[15] W. Shi, J. Wu, B.P. Rosen, Identification of a putative metal binding site in a new family of metalloregulatory proteins, J Biol Chem, 269 (1994) 19826-9.

[16] C. Xu, B.P. Rosen, Dimerization is essential for DNA binding and repression by the ArsR metalloregulatory protein of Escherichia coli, J Biol Chem, 272 (1997) 15734-8.

[17] D.R. Campbell, K.E. Chapman, K.J. Waldron, S. Tottey, S. Kendall, G. Cavallaro, C. Andreini, J. Hinds, N.G. Stoker, N.J. Robinson, J.S. Cavet, Mycobacterial cells have dual nickel-cobalt sensors: sequence relationships and metal sites of metal-responsive repressors are not congruent, J Biol Chem, 282 (2007) 32298-310.

[18] C. Darnault, A. Volbeda, E.J. Kim, P. Legrand, X. Vernede, P.A. Lindahl, J.C. Fontecilla-Camps, Ni-Zn-[Fe4-S4] and Ni-Ni-[Fe4-S4] clusters in closed and open subunits of acetyl-CoA synthase/carbon monoxide dehydrogenase, Nat Struct Biol, 10 (2003) 271-9.

[19] Y. Li, D.B. Zamble, Nickel homeostasis and nickel regulation: an overview, Chem Rev, 109 (2009) 4617-43.

[20] U. Kretzschmar, M. Schobert, H. Gorisch, The Pseudomonas aeruginosa acsA gene, encoding an acetyl-CoA synthetase, is essential for growth on ethanol, Microbiology, 147 (2001) 2671-7.

[21] P. Teriete, Y. Yao, A. Kolodzik, J. Yu, H. Song, M. Niederweis, F.M. Marassi, Mycobacterium tuberculosis Rv0899 adopts a mixed alpha/beta-structure and does not form a transmembrane beta-barrel, Biochemistry, 49 (2010) 2768-77.

[22] I. Hanukoglu, Proteopedia: Rossmann fold: A beta-alpha-beta fold at dinucleotide binding sites, Biochem Mol Biol Educ, 43 (2015) 206-9.

[23] E. Skovran, D.M. Downs, Lack of the ApbC or ApbE protein results in a defect in Fe-S cluster metabolism in Salmonella enterica serovar Typhimurium, J Bacteriol, 185 (2003) 98-106.

[24] J.E. Pitzer, S.Z. Sultan, Y. Hayakawa, G. Hobbs, M.R. Miller, M.A. Motaleb, Analysis of the Borrelia burgdorferi cyclic-di-GMP-binding protein PlzA reveals a role in motility and virulence, Infect Immun, 79 (2011) 1815-25.

[25] A.J. Wolfe, K.L. Visick, Get the message out: cyclic-Di-GMP regulates multiple levels of flagellum-based motility, J Bacteriol, 190 (2008) 463-75.

[26] A. Lewit-Bentley, S. Rety, EF-hand calcium-binding proteins, Curr Opin Struct Biol, 10 (2000) 637-43.

[27] T. Schmidt, H.G. Schlegel, Nickel and cobalt resistance of various bacteria isolated from soil and highly polluted domestic and industrial wastes, FEMS Microbiology Letters, 62 (1989) 315-328.

[28] V. Srikannathasan, G. English, N.K. Bui, K. Trunk, P.E. O'Rourke, V.A. Rao, W. Vollmer, S.J. Coulthurst, W.N. Hunter, Structural basis for type VI secreted peptidoglycan DL-endopeptidase function, specificity and neutralization in Serratia marcescens, Acta Crystallogr D Biol Crystallogr, 69 (2013) 2468-82.

[29] C. Yeats, N.D. Rawlings, A. Bateman, The PepSY domain: a regulator of peptidase activity in the microbial environment?, Trends Biochem Sci, 29 (2004) 169-72.

**Table S1**

|  | **Sequenced fragments** | **Mapped fragments** |
| --- | --- | --- |
| **LB culture 1** | 2510606 | 2241487 |
| **LB culture 2** | 2450749 | 2163115 |
| **LB culture 3** | 2902870 | 2580290 |
| **LB-Ni culture 1** | 1792002 | 1599543 |
| **LB-Ni culture 2** | 1618659 | 1470918 |
| **LB-Ni culture 3** | 1513739 | 1329514 |

**Table S1. Sequenced cDNA fragments and their mapping.** The number of sequenced and quality-checked cDNA fragments for each RNA preparation is reported. The number of fragments mapped on the *Sphingobium* sp. ba1 genome is also given.

| **Table S2** | | | | | | | | | | |
| --- | --- | --- | --- | --- | --- | --- | --- | --- | --- | --- |
|  | **Contig** | **Acc. num.** | **NCBI Annotation** | **Protein Length** | **CDD** | | **Fold change** | **Operon*** | **Cellular Process^1^** | **Cellular Component^2^** |
| I.i | JPPQ01000083.1.248 | IL54_0273 | Hypothetical protein (Hp) | 90 |  | | 2.28 | 8/8 Op. 63 | U | U |
|  | JPPQ01000083.1.249 JPPQ01000083.1.250 | IL54_0274 IL54_0275 | Hp Hp | 189 219 | CDD:258078_HupE/UreJ(33-185) | | 7.32 | 6-7/8 Op. 63 | U U | U U |
|  | JPPQ01000083.1.251 | IL54_0277 | Hp | 140 |  | | 5.22 | 5/8 Op. 63 | U | U |
|  | JPPQ01000083.1.252 JPPQ01000083.1.253 JPPQ01000083.1.254 | IL54_0278 IL54_0279 IL54_0280 | Cu/Ag efflux system CusA/CzcA MFP Hp | 1063 384 422 | CDD:224455_TolC (47-413) | | 5.14 | 2-4/8 Op. 63 | T T T | M M U |
|  | JPPQ01000083.1.256 | IL54_0282 | Hp | 418 | CDD:119392_MFS(1-372) | | 5.23 | 1/8 Op. 63 | T | U |
|  | JPPQ01000083.1 | IL54_4659 | Hp | 110 |  | | 5.20 |  | U | U |
|  | JPPQ01000069.1.1134 | IL54_1860 | putative membrane protein | 156 |  | | 3.24 |  | U | U |
|  | JPPQ01000069.1.1136 | IL54_1862 | putative membrane protein | 228 |  | | 1.25 |  | U | U |
| I.ii | JPPQ01000083.1.64 | IL54_0078 | NAD-dependent aldehyde dehydrogenase | 506 |  | | 2.20 | 1/6 Op. 17 | Ox | U |
|  | JPPQ01000083.1.65 | IL54_0079 | Zn-dependent alcohol dehydrogenase | 341 |  | | 3.36 | 2/6 Op. 17 | Ox | U |
|  | JPPQ01000083.1.66 JPPQ01000083.1.67 JPPQ01000083.1.68 | IL54_0080 IL54_0081 IL54_0082 | copper resistance protein CopA copper r. p. CopB putative MFS permease | 568 382 378 |  | | 3.81 | 3-5/6 Op. 17 | Ox O T | P M M |
|  | JPPQ01000069.1.1543 JPPQ01000069.1.1544 | IL54_2270 IL54_2271 | CopB Putative multicopper oxidase | 301 650 | CDD:273649_CopA (4-644) | | 2.24 | 1-2/2 Op. 359 | O Ox | U P |
|  | JPPQ01000069.1.1548 | IL54_2275 | copper resistance protein CopC | 125 | CopC domain | | 5.93 | 1/3 Op. 361 | O | P |
|  | JPPQ01000011.1.1 | IL54_4660 | CopA (part) |  |  | | 1.16 |  |  |  |
|  | JPPQ01000083.1.171 | IL54_0192 | cation transport ATPase | 697 | CDD:273664_ATPase-IB1 family | | 1.64 |  | T | M |
| I.iii | JPPQ01000069.1.2068 JPPQ01000069.1.2069 | IL54_2795 IL54_2796 | Hp Hp | 363 493 | CDD:224753_ABC-type Fe3+ transport system (62-361) CDD:276322:OM_channels (118-493) | | 3.12 | 1-2/3 Op. 473 | U U | U U |
|  | JPPQ01000069.1.2070 | IL54_2797 | Hp | 433 | CDD: 225407 CitM H+/citrate symporter 3-431) | | 4.22 | 3/3 Op. 473 | T | M |
|  | JPPQ01000069.1.2461 | IL54_3183 | Hp | 115 | CDD:275226_UrcA family (Uranium responsive) (21-93) | | 1.17 |  | U | U |
|  | JPPQ01000069.1.2546 | IL54_3276 | bacterioferritin | 159 |  | | 1.70 |  | T | C |
| I.iv | JPPQ01000083.1.432 | IL54_0463 | TonB-dependent receptor-like protein | 702 |  | | 2.60 | 1/3 Op. 432 | T | M |
|  | JPPQ01000083.1.433 JPPQ01000083.1.434 | IL54_0464 IL54_0465 | Hp Putative iron-uptake protein | 188 456 |  | | 5.02 | 2-3/3 Op. 432 | U U | U U |
|  | JPPQ01000069.1.1116 | IL54_1841 | Hp | 700 | CDD:238657 TonB-dependent ligand_gated_channel (59-692) | | 2.02 | 1/2 Op. 257 | T | M |
|  | JPPQ01000069.1.2264 | IL54_2990 | TonB-like protein | 205 |  | | 2.00 |  | T | U |
|  | JPPQ01000069.1.2265 | IL54_2991 | iron complex outermembrane receptor protein | 792 |  | | 2.20 |  | T | M |
|  | JPPQ01000069.1.2328, JPPQ01000069.1.2329 | IL54_3055 IL54_3056 | Hp Hp | 211 183 | CDD:257540_PepSY__TM_I (4-31) CDD:258004_PepSY_TM_III (10-46) | | 1.87 | 2/3, 3/3 Op. 526 | U U | U U |
|  | JPPQ01000069.1.2330 | IL54_3057 | TonB-dependent receptor-like protein | 671 |  | | 2.25 | 1/3 Op. 526 | T | M |
|  | JPPQ01000023.1.4 | IL54_4442 | Hp | 98 | CDD:277538_OM_Channels (<2-98) | | 3.54 |  | T | M |
|  | JPPQ01000059.1.1 | IL54_4661 |  | 250 | part of a TonB-dependent siderophore receptor of about 715 aa. | | 1.30 |  |  |  |
| I.v | JPPQ01000083.1.545 | IL54_0580 | HlyD-family secretion protein | 370 |  | | 3.40 | 1/3 Op. 139 | T | M |
|  | JPPQ01000083.1.546 JPPQ01000083.1.547 | IL54_0581 IL54_0582 | HAE1-fam efflux prot RND-fam efflux transporter | 1051 454 |  | | 2.97 | 2-3/3 Op. 139 | T T | M M |
|  | JPPQ01000083.1.165 JPPQ01000083.1.166 | IL54_0186 IL54_0187 | L-serine dehydratase Hp | 316 169 | CDD:224455_Outer membrane efflux protein Tolc (<2-169) | | 1.63 | 3-4/4 op. 43 | O T | U U |
| II | JPPQ01000008.1.1 | IL54_4429 | Hp | 53 | CDD:263790_Catalase-like heme-binding protein (<2-36) | | 2.72 | 1/3 Op. 1 | Ox | U |
|  | JPPQ01000008.1.2 JPPQ01000008.1.3 | IL54_4430 IL54_4431 | Hp Hp | 205 455 | CDD:275695_Dyp-type peroxidase family (19-438) | | 2.12 | 2-3/3 Op. 1 | U Ox | U U |
|  | JPPQ01000069.1.866 | IL54_1589 | putative transposase | 408 | CDD:163708_Catalase-like heme-binding proteins similar to the uncharacterized y4iL (43-215) | | 2.41 |  | Ox | U |
|  | JPPQ01000069.1.929 | IL54_1653 | Hp | 351 | CDD:163708_Catalase-like heme-binding proteins similar to the uncharacterized y4iL; | | 1.91 |  | Ox | U |
|  | JPPQ01000087.1.1 | IL54_0657 | Hp | >58 | CDD:250086_3-hydroxyacyl-CoA dehydrogenase (<1-54) | | 1.92 | 1/2 Op. 1 | Ox | U |
|  | JPPQ01000087.1.2 | IL54_4662 | Hp | >450 | The encoded polypeptide (about 450 aa) is identical to 67 kDa myosin-cross-reactive antigen-like protein of Novosphingobium resinovorum | | 1.60 | 2/2 Op. 1 |  |  |
|  | JPPQ01000069.1.1576 | IL54_2303 | putative hydroxylase | 109 | CDD:276328_2OG-FeII_Oxy oxygenase superfamily (<2-109) | | 1.98 |  | Ox | U |
|  | JPPQ01000069.1.3079 | IL54_3810 | methylmalonate-semialdehyde dehydrogenase | 499 |  | | 1.29 | 1/3 Op. 697 | Ox | U |
| III | JPPQ01000083.1.257 | IL54_0283 | Hp | 61 (89) | CDD:260655_CsoR_like_DUF156 (<1-61) | | 3.14 |  | R | U |
|  | JPPQ01000069.1.42 JPPQ01000069.1.43 | IL54_0770 IL54_0771 | signal transduction his kinase response regulator | 455 242 |  | | 1.57 | 1/2, 2/2 Op. 11 | R R | M I |
|  | JPPQ01000069.1.67 | IL54_0796 | Hp | 457 | CDD:275695_Dyp-type peroxidase family (19-438) | | 1.86 | 3/3 Op. 18 | Ox | U |
|  | JPPQ01000069.1.69 JPPQ01000069.1.70 | IL54_0797 IL54_0798 | Hp ArsR-family transcriptional regulator | 115 112 |  | | 1.63 | 1-2/3 Op. 18 | U R | U U |
|  | JPPQ01000069.1.1027 JPPQ01000069.1.1028 | IL54_1746 IL54_1747 | GntR-family transcript. reg. Hp | 197 309 | CDD:276328_2OG-FeII_Oxy (22-205) | | 3.88 | 1-2/2 Op. 236 | R U | U U |
| IV | JPPQ01000083.1.247 | IL54_0272 | Hp | 523 | CDD:249567_Reverse transcriptase (52-275); CDD:257693_Trypsin_2 (365-490). | | 1.99 |  | O | U |
|  | JPPQ01000083.1.503 | IL54_0539 | acetyl-CoA synthetase | 647 |  | | 1.33 |  | O | U |
|  | JPPQ01000070.1.20 | IL54_0678 | ABC-type transport system permease component | 401 |  | | 4.02 |  | U | M |
|  | JPPQ01000069.1.218 | IL54_0951 | OmpA_C-like | 255 |  | | 1.48 |  | U | M |
|  | JPPQ01000069.1.297 | IL54_1032 | Hp | 184 | CDD:273591_Intracellular protease PfpI family (9-176) | | 4.58 |  | O | U |
|  | JPPQ01000069.1.933 | IL54_1658 | Hp | 356 | CDD:277520_NADB Rossmann | | 1.65 | 4/4 Op. 214 | O | U |
|  | JPPQ01000069.1.2006 | IL54_2731 | ferredoxin | 146 | CDD:153434_Cystein-rich four helical bundle bacterial protein (39-141) | | 3.26 | 9/9 Op. 459 | U | U |
|  | JPPQ01000069.1.2123 | IL54_2853 | outer membrane protein W | 220 |  | | 4.43 |  | U | M |
|  | JPPQ01000069.1.2729 | IL54_3467 | thiamine biosynthesis lipoprotein | 289 |  | | 2.54 |  | O | U |
|  | JPPQ01000006.1.176 | IL54_4207 | putative beta-lactamase class A | 353 |  | | 1.42 |  | U | U |
| V | JPPQ01000083.1.159 | IL54_0179 | Hp | 122 | CDD:275707_PilZ Domain (32-100) | | 1.51 |  | U | U |
|  | JPPQ01000069.1.4 | IL54_0733 | Hp | 73 | CDD:277520_NADB Rossmann | | 1.70 | 2/2 di Op. 1 | U | U |
|  | JPPQ01000069.1.181 | IL54_0911 | Hp | 243 | CDD:260779_Protein of unknown function (DUF541) (29-243) | | 1.20 | 1/5 Op. 47 | U | U |
|  | JPPQ01000069.1.264 | IL54_0998 | Hp | 167 | CDD:147887_Domain of Unknown Function (DUF892) (6-165) | | 1.32 | 2/6 Op. 64 | U | U |
|  | JPPQ01000069.1.535 | IL54_1269 | Hp | 130 | Possible Fe-S oxidoreductase | | 1.55 |  | U | U |
|  | JPPQ01000069.1.684 | IL54_1413 | Hp | 148 | CDD:277449_EF-hand, calcium binding motif (78-140) | | 2.45 |  | U | U |
|  | JPPQ01000069.1.1117 | IL54_1842 | Hp | 263 | CDD:256113_Domain of unknown function (DUF4198) (21-241) | | 3.01 | 1/2 Op.258 | U | U |
|  | JPPQ01000069.1.1118 | IL54_1843 | Hp | 165 |  | | 2.21 | 2/2 Op.258 | U | U |
|  | JPPQ01000069.1.1302 | IL54_2025 | Hp | 63 |  | | 1.66 |  | U | U |
|  | JPPQ01000069.1.1907 | IL54_2636 | Hp | 87 | CDD:276033_Cystein-rich four helical bundle bacterial protein (<1-81) | | 3.96 |  | U | U |
|  | JPPQ01000069.1.2120 | IL54_2850 | Hp | 120 |  | | 1.79 |  | U | U |
|  | JPPQ01000069.1.2173 | IL54_2900 | Hp | 400 |  | | 1.63 |  | U | U |
|  | JPPQ01000069.1.2349 | IL54_3074 | Hp | 191 | CDD:253528_Domain of unknown function (DUF924) (19-189) | | 1.27 |  | U | U |
|  | JPPQ01000069.1.2726 | IL54_3464 | Hp | 214 | CDD:225832_Uncharacterized protein | | 2.66 | 1/3 Op. 612 | U | U |
|  | JPPQ01000069.1.2727 | IL54_3465 | Hp | 177 | CDD:276354_FlgD Ig-like domain | | 2.09 | 2/3 Op. 612 | U | U |
|  | JPPQ01000069.1.2728 | IL54_3466 | Hp | 302 | CDD:256113_Domain of unknown function (DUF4198) (20-245) | | 1.33 | 3/3 Op. 612 | U | U |
|  | JPPQ01000069.1.2881 | IL54_3615 | Hp | 93 |  | | 2.18 |  | U | U |
|  | JPPQ01000049.1.9 | IL54_4408 | Hp | 38 |  | | 1.44 | 1/3 Op. 8 | U | U |
|  | JPPQ01000034.1.9 | IL54_4421 | Hp | 126 |  | | 1.13 | 1/4 Op. 3 | U | U |
|  | JPPQ01000006.1.244 | IL54_4274 | Hp | 283 | CDD:255769_Predicted metal-dependent hydrolase (8-259) | | 1.71 | 1/2 Op. 53 | U | U |
|  | JPPQ01000083.1.61 | IL54_4663 |  | 291 |  | | 2.05 |  |  |  |
|  | JPPQ01000083.1.62 | IL54_4664 |  | 402 |  | | 1.53 | 1/2 Op. 16 |  |  |
| **Down-regulated genes** | JPPQ01000083.1.29 | IL54_0041 | Hp | 102 |  | | -1.51 |  | U | U |
|  | JPPQ01000069.1.153 | IL54_0881 | Hp | 555 | CDD:223824_Catalase [Inorganic ion transport and metabolism] (67-529) | | -1.18 |  | Ox | U |
|  | JPPQ01000069.1.315 | IL54_1050 | Hp | 879 | CDD:224544_CirA OM receptor protein (1-879) | | -1.25 |  | T | M |
|  | JPPQ01000069.1.481 | IL54_1211 | putative oxidase | 504 |  | | -1.19 |  | U | U |
|  | JPPQ01000069.1.825 | IL54_1553 | phage shock protein A | 222 |  | | -1.25 |  | U | U |
|  | JPPQ01000069.1.1172 | IL54_1900 | LysE-family transporter | 204 |  | | -1.31 |  | T | M |
|  | JPPQ01000069.1.1280 | IL54_2004 | TonB_dependent receptor-like | 847 |  | | -2.00 |  | T | M |
|  | JPPQ01000069.1.1386 | IL54_2111 | polysaccharide export outer membrane protein | 214 |  | | -1.56 |  | T | M |
|  | JPPQ01000069.1.1562 | IL54_2289 | Hp | 137 |  | | -1.30 | 1/4 Op. 366 | U | U |
|  | JPPQ01000069.1.1563 | IL54_2290 | TonB-dependent receptor-like protein | 676 |  | | -4.35 | 2/4 Op. 366 | T | M |
|  | JPPQ01000069.1.1564 | IL54_2291 | Hp | 335 | CDD:225723_PiuB (5->259) | | -3.52 | 3/4 Op. 366 | U | U |
|  | JPPQ01000069.1.1929 | IL54_2659 | pirin-related protein | 239 | CDD:Redox-sensitive bicupin YhaK (9-237) | | -1.23 |  | U | U |
|  | JPPQ01000069.1.2088 | IL54_2819 | flagellar hook-associated protein 2 | 462 |  | | -1.41 | 1/2 Op. 477 | O | F |
|  | JPPQ01000069.1.2089 | IL54_2820 | flagellin-specific chaperone FliS | 131 |  | | -1.64 | 2/2 Op. 477 | U | F |
|  | JPPQ01000069.1.2291 | IL54_3017 | Hp | 66 | CDD:148310_DUF 1153 (part <2-57) | | -1.30 |  | U | U |
|  | JPPQ01000069.1.2408 | IL54_3131 | Flp pilus assembly protein CpaB | 344 |  | | -1.20 |  | U | U |
|  | JPPQ01000069.1.2486 JPPQ01000069.1.2487 | IL54_3208 IL54_3209 | Hp putative dehydrogenase | 179 540 | CDD:225723_Unch. Fe-regulated memb. protein(<10-173) | | -1.92 | 1-2/2 Op. 558 | U U | U U |
|  | JPPQ01000069.1.2840 | IL54_3574 | flagellar basal-body rod protein FlgF | 247 |  | | -1.32 |  | U | U |
|  | JPPQ01000069.1.2855 | IL54_3590 | flagellin | 248 |  | | -2.02 |  | O | F |
|  | JPPQ01000069.1.2856 | IL54_3591 | CheY-like response regulator | 395 |  | | -1.34 |  | R | I |
|  | JPPQ01000069.1.3127 | IL54_3857 | ribosomal protein L17 | 137 |  | | -1.15 |  | O | R |
|  | JPPQ01000028.1.4 | IL54_0003 | Hp | 173 | CDD:277532_MFS (<2-153) | | -1.51 |  | U | U |
|  | JPPQ01000069.1.3186 | IL54_3918 | arabinose polymer transporter | 398 | Belongs to MFS family | | -1.34 |  | T | M |
|  | JPPQ01000006.1.218 | IL54_4247 | Hp | 106 |  | | -1.80 | 1/2 Op. 45 | U | U |
|  | JPPQ01000006.1.254 | IL54_4285 | ribosomal protein L1 | 232 |  | | -1.27 | 5/6 Op. 56 | O | R |
|  | JPPQ01000006.1.255 | IL54_4286 | ribosomal protein L11 | 111 |  | | -1.25 | 4/6 Op. 56 | O | R |
|  | JPPQ01000006.1.287 | IL54_4319 | Hp | 251 |  | | -1.15 |  | U | U |
| **Table S2. Differentially expressed genes in Ni exposed *Shingobium* sp. ba1 cells**. Up-regulated genes are subdivided into five sections (I-V) according to their description in the Result and Supplementary Material sections. For each gene the table reports the accession number of the related contig, the accession number (corresponding to the gene in the European Bioinformatics Institute (EBI) database), the description and the length of the encoded protein, the ID and description of the identified CDD (mainly for uncharacterized proteins), the expression level fold change, the corresponding operon as identified by EuGene analysis, and the cellular process and component reported in the Uniprot database (http://www.uniprot.org). Genes belonging to the same operon are highlighted in gray in the Operon column. Gene reported in red correspond to core genes. | | | | | | | | | | |
|  |  |  |  |  |  |  |  |  |  |  |
| *) The operon number is as reported in the EuGene data. The position of the gene (x) with respect to the number (y) of genes in the operon is given as x/y. Operons with more than one gene differentially expressed are reported with gray background. | | | | | | | | | | |
| 1) The letter code is: U = Uncharacterized process; T = Transport and homeostasis; Ox = Oxidative-Reductive process; R = Regulation og gene expression; O = Other process. | | | | | | | | | | |
| 2) The letter code is: U = uncharacterized Cellular component; M = Membrane; P = Periplasm; I = Intracitoplasmic; R = Ribosome. | | | | | | | | |  |  |

**Table S3**

| S. sp. ba1 | IL54_0273 | IL54_0274 | IL54_0275 | **IL54_0277** | **IL54_0278** | **IL54_0279** | **IL54_0280** | IL54_4659 | **IL54_0282** | **IL54_0283** |
| --- | --- | --- | --- | --- | --- | --- | --- | --- | --- | --- |
| yanoikuyae SHJ | **+** | **+** | **+** | **+** | **+** | **+** | **+** | **+** | **+** | **+** |
| yanoikuyae ATCC 51230 | **+** | **+** | **+** | **+** | **+** | **+** | **+** | **+** | **+** | **+** |
| yanoikuyae TJ | **+** | **+** | **+** | **-** | **+** | **+** | **+** | **+** | **+** | **+** |
| yanoikuyae B1 Malaysia | **-** | **-** | **-** | **-** | **+** | **+** | **+** | **+** | **+** | **+** |
| Ant17 | **-** | **+** | **+** | **-** | **-** | **+** | **-** | **-** | **-** | **-** |
| chungbukense | **+** | **+** | **+** | **-** | **-** | **-** | **-** | **-** | **-** | **-** |
| lactosensus | **-** | **-** | **-** | **-** | **+** | **-** | **-** | **-** | **+** | **+** |
| C100 | **-** | **-** | **-** | **-** | **-** | **-** | **-** | **-** | **-** | **-** |
| Leaf26 | **-** | **-** | **-** | **-** | **-** | **-** | **-** | **-** | **-** | **-** |
| baderi | **-** | **-** | **-** | **-** | **-** | **+** | **-** | **-** | **-** | **-** |
| SYK-6 | **-** | **+** | **+** | **-** | **-** | **-** | **-** | **-** | **-** | **-** |
| YBL2 | **-** | **-** | **+** | **-** | **-** | **-** | **-** | **-** | **-** | **-** |
| DC-2 | **-** | **-** | **-** | **-** | **-** | **-** | **-** | **-** | **-** | **-** |
| YL23 | **-** | **-** | **-** | **-** | **-** | **-** | **-** | **-** | **-** | **-** |
| KK22 | **-** | **-** | **-** | **-** | **-** | **-** | **-** | **-** | **-** | **-** |
| xenophagum QQY | **-** | **-** | **-** | **-** | **-** | **-** | **-** | **-** | **-** | **-** |
| AP49 | **-** | **-** | **-** | **-** | **-** | **-** | **-** | **-** | **-** | **-** |
| chinhatense | **-** | **-** | **-** | **-** | **-** | **-** | **-** | **-** | **-** | **-** |
| chlorophenolicum | **-** | **-** | **-** | **-** | **-** | **-** | **-** | **-** | **-** | **-** |
| HDIP04 | **-** | **-** | **-** | **-** | **-** | **-** | **-** | **-** | **-** | **-** |
| herbicidovorans | **-** | **-** | **-** | **-** | **-** | **-** | **-** | **-** | **-** | **-** |
| indicum | **-** | **-** | **-** | **-** | **-** | **-** | **-** | **-** | **-** | **-** |
| Japonicum UT26S | **-** | **-** | **-** | **-** | **-** | **-** | **-** | **-** | **-** | **-** |
| lucknowense | **-** | **-** | **-** | **-** | **-** | **-** | **-** | **-** | **-** | **-** |
| quisquilarum | **-** | **-** | **-** | **-** | **-** | **-** | **-** | **-** | **-** | **-** |
| unmariense | **-** | **-** | **-** | **-** | **-** | **-** | **-** | **-** | **-** | **-** |
| xenophagum NBRC 107872 | **-** | **-** | **-** | **-** | **-** | **-** | **-** | **-** | **-** | **-** |
| yanoikuyae B1 Shangai | **-** | **-** | **-** | **-** | **-** | **-** | **-** | **-** | **-** | **-** |
| yanoikuyae XLDN2-5 | **-** | **-** | **-** | **-** | **-** | **-** | **-** | **-** | **-** | **-** |

**Table S3. Comparative Genomics of *Shingobium* sp. ba1 genes IL54_0273- IL54_0283 in complete genomes *of S. yanoikuyae* species.** Conserved genes are indicated by + sign; partially conserved and absent genes are indicated by * and – symbols, respectively. Genes IL54_0273- IL54_0282 constitute the operon n. 63 of contig JPPQ01000083. The IL54_0283 gene is adjacent to the operon.

**Table S4**

| ***Sphingobium* sp. ba1** | | | ***S. yanoikuyae*** | | | | | | | |
| --- | --- | --- | --- | --- | --- | --- | --- | --- | --- | --- |
|  |  |  | **ATCC_51230** | | | | **SHJ** | | | |
| **Protein a.n.** | **length** | **Annotation** | **Protein a.n.** | **length** | **Annotation** | **Similarity %** | **Protein a.n.** | **length** | **Annotation** | **Similarity %** |
| KFL44906  ( IL54_0273) | 90 | Hypothetical protein (Hp) | EKU74187 | 89 | Hp | 98.9 | BV87_19905 | 89 | Hp | 98.9 |
| KFL44907  ( IL54_0274) | 189 | Hp | EKU74183 | 196 | Hp | 96.4 | BV87_19910 | 196 | Hp | 96.4 |
| KFL44908  ( IL54_0275) | 219 | Hp | EKU74182 | 237 | Hp | 92.0 | BV87_19915 | 219 | Membrane protein | 99.5 |
| KFL44910  ( IL54_0277) | 140 | Hp | EKU74181 | 158 | Hp | 88.6 | BV87_19925 | 158 | Hp | 88.6 |
| KFL44911  ( IL54_0278) | 1063 | Cu/Ag efflux system CusA/CzcA | EKU74180 | 1078 | CzcA family heavy metal efflux pump | 98.6 | BV87_19930 | 1078 | Cation transporter | 98.6 |
| KFL44912  ( IL54_0279) | 384 | MFP | EKU74179 | 384 | Efflux transporter, RND family, MFP subunit | 100.0 | BV87_19935 | 384 | Metal transporter | 100.0 |
| KFL44913  ( IL54_0280) | 422 | Hp | EKU74178 | 422 | Hp | 100.0 | BV87_19940 | 422 | Metal transporter | 100.0 |
| N.A.  ( IL54_4659) | 109 | Hp | N.A. | 109 | Hp | 100.0 | BV87_19945 | 110 | Hp | 99.1 |
| KFL44915  ( IL54_0282) | 418 | Hp | EKU74177 | 445 | H^+^ Antiporter protein | 93.7 | BV87_19950 | 445 | Major facilitato transporter | 93.7 |
| KFL44916  ( IL54_0283) | 61 (89)* | Hp | EKU74176 | 89 | Hp | 98.9 | BV87_19955 | 89 | NreA | 98.9 |

**Table S4.** Similarity values between proteins encoded by genes IL54_0274-IL54_0283 (including IL54_4659) in *Sphingobium* sp. ba1 contig JPPQ01000083.1 and homologous counterparts in the *S. yanoikuyae* strains ATCC_51230 (contig AGZU01000009) and SHJ (contig JFFT01000049).

N.A. = Not available

*) The length between brackets is derived from EuGene transcriptome analysis.

**Figure S1**

Figure S1. RT-PCR analysis of the JPPQ01000083-63 operon genes. Fold-changes of expression for genes in putative operon 63 between control and LB-Ni culture as evaluated by RT-PCR (violet) and RNA-seq (red).

**Figure S2**


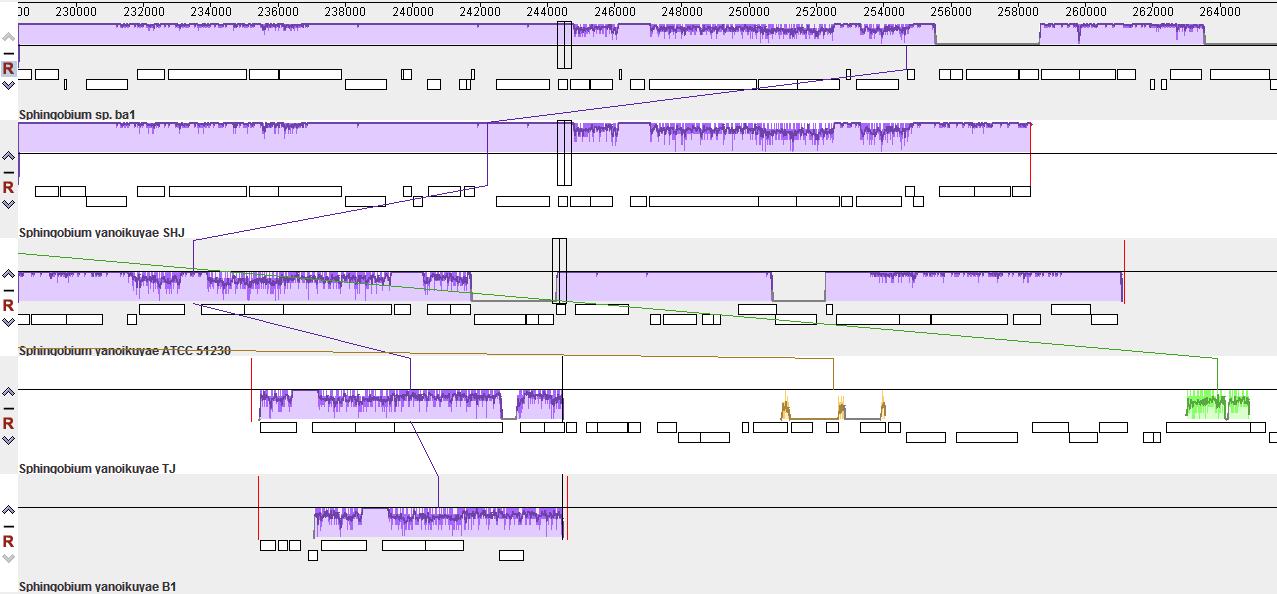


**1 2 3 4 5 6 7 8 9 10**

**1 2 3 4 5 6 7 8 9 10**

**10 9 8 7 6 5 4 3 2 1**

**9 8 7 6 3 2**

**10 9 8 7 6 5**

**IL54_0273**

**IL54_0274**

**IL54_0275**

**IL54_0277**

**IL54_0278**

**IL54_0279**

**IL54_0280**

**IL54_4659**

**IL54_0282**

**IL54_0283**

Figure S2. MAUVE comparison of IL54_0274-IL54_0283 cluster with a selection of *S. yanoikuyae* genomic regions*.* Homologous genes are indicated by red numbers. Genomes alignment has been obtained in relation to gene IL54_0274 (double vertical rectangles). Genome alignments were generated using the “progressive alignment” option available in the MAUVE program with default scoring and parameters.

**Figure S3**


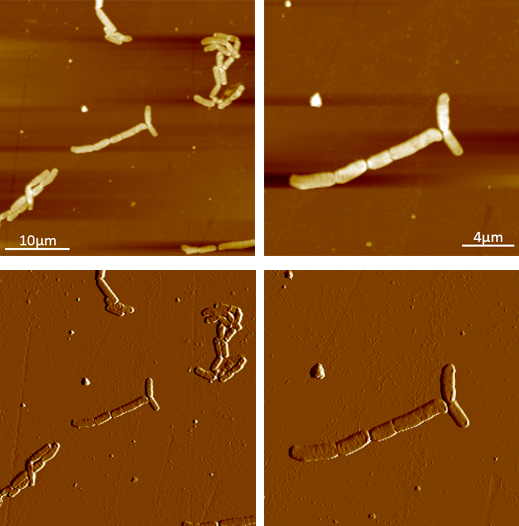

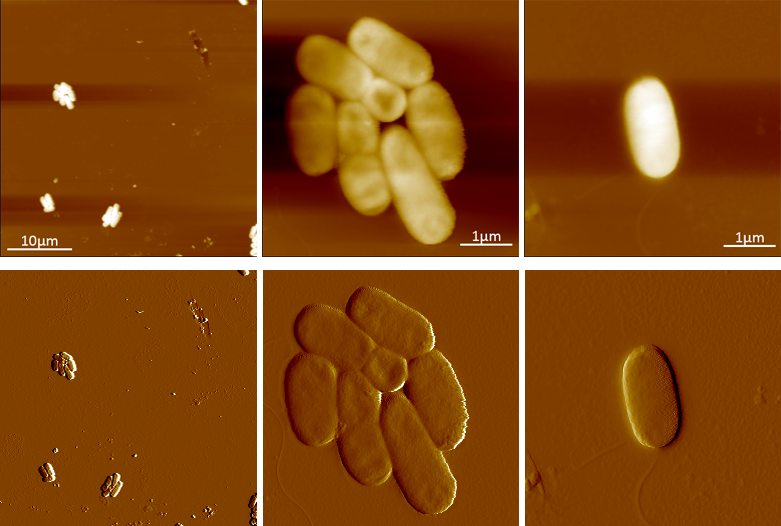

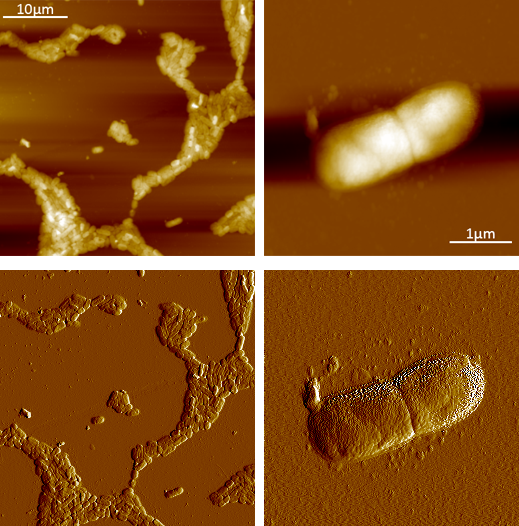

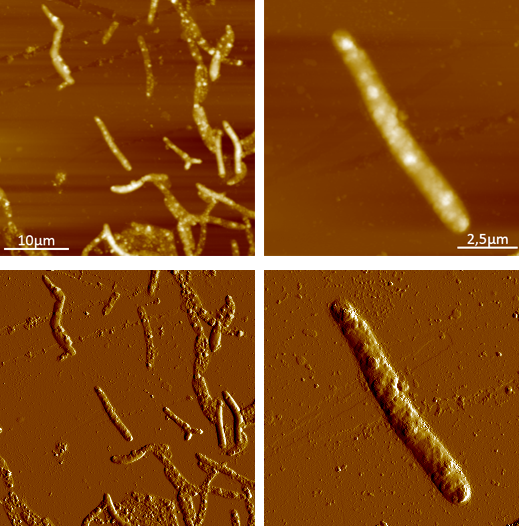


Figure S3. AFM images of a *Sphingobium* sp. ba1 cells cultured in LB (first two lines) and LB-Ni media (third and fourth lines). Images were taken in topographic and amplitude modes (odd and even lines, respectively).

**Figure S4**

.
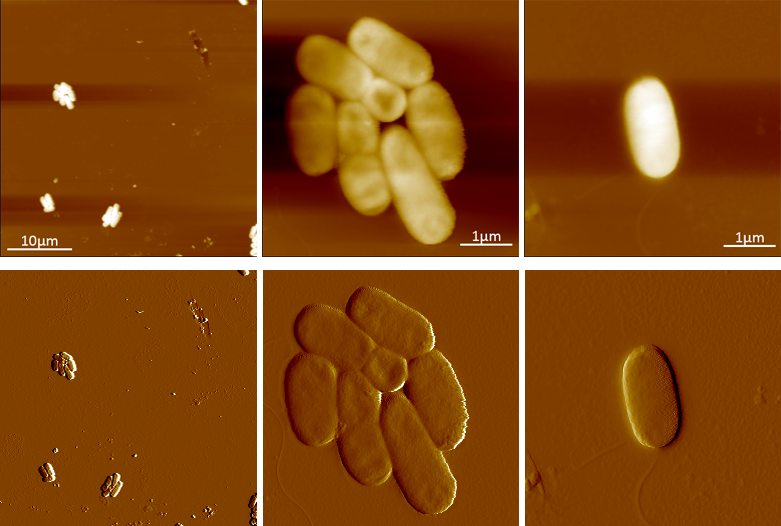

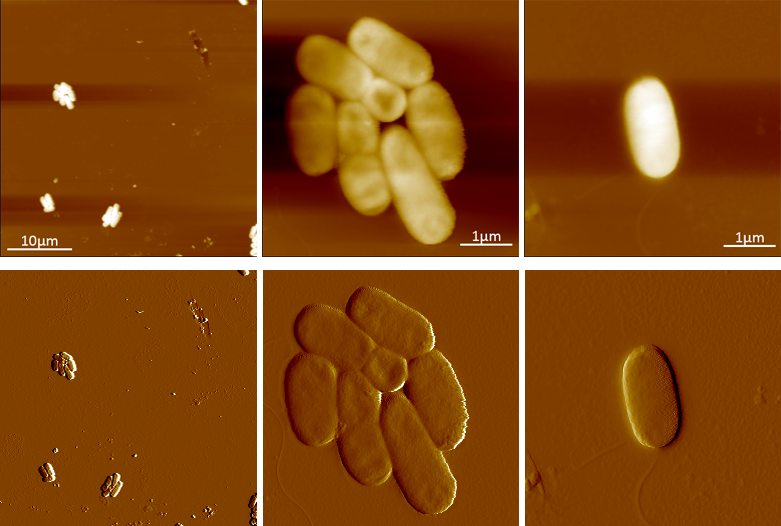


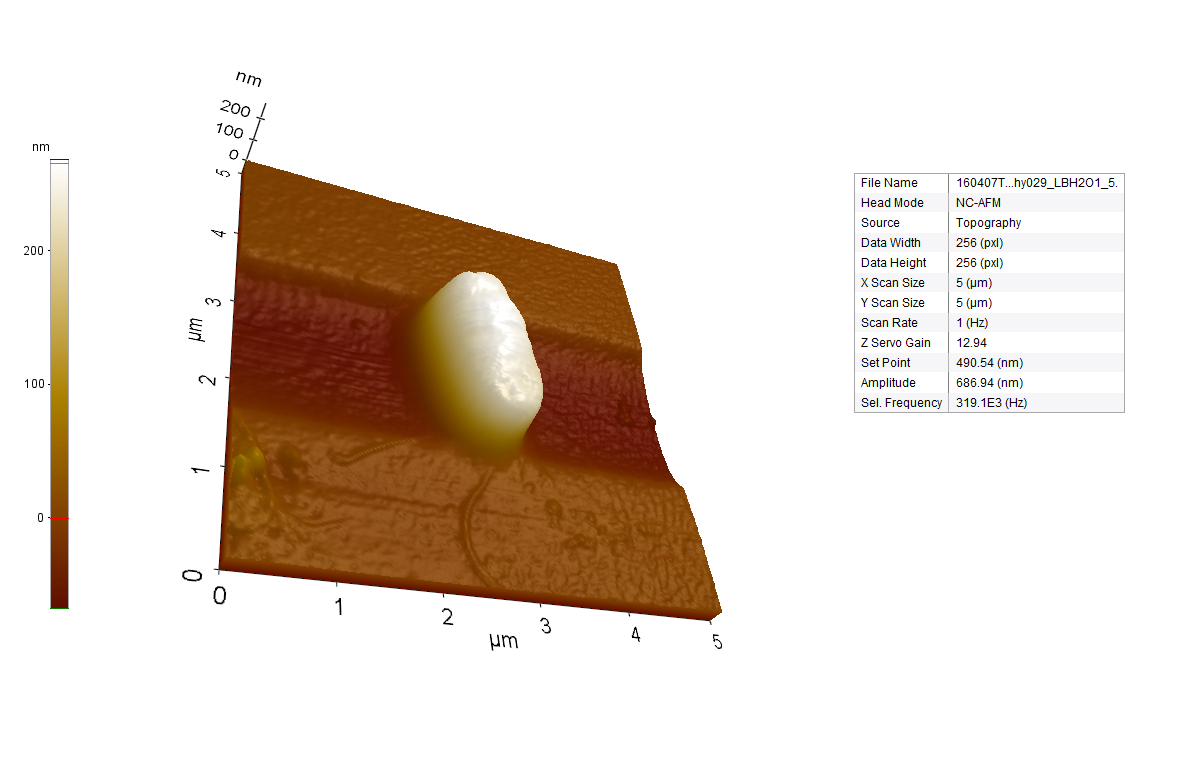


Figure S4. AFM images of a *Sphingobium* sp. ba1 cell cultured in LB medium (clockwise from top-left: topographic, amplitude and 3D modes), showing the presence of possible flagella.
